# Correlation Between Antimicrobial Structural Classes and Membrane Partitioning: Role of Emerging Lipid Packing Defects

**DOI:** 10.1007/s00232-024-00318-z

**Published:** 2024-07-22

**Authors:** S. V. Sankaran, Roni Saiba, Samapan Sikdar, Satyavani Vemparala

**Affiliations:** 1 The Institute of Mathematical Sciences, C.I.T. Campus, Taramani, Chennai, Tamil Nadu 600113 India; 2 Homi Bhabha National Institute, Training School Complex, Anushakti Nagar, Mumbai, Maharashtra 400094 India

**Keywords:** Antimicrobial peptides, Bacterial membrane, MD simulation

## Abstract

In this study, a combination of bioinformatics and molecular dynamics simulations is employed to investigate the partitioning behavior of different classes of antimicrobial peptides (AMPs) into model membranes. The main objective is to identify any correlations between the structural characteristics of AMPs and their membrane identification and early-stage partitioning mechanisms. The simulation results reveal distinct membrane interactions among the various structural classes of AMPs, particularly in relation to the generation and subsequent interaction with lipid packing defects. Notably, AMPs with a structure-less coil conformation generate a higher number of deep and shallow defects, which are larger in size compared to other classes of AMPs. AMPs with helical component demonstrated the deepest insertion into the membrane. On the other hand, AMPs with a significant percentage of beta sheets tend to adsorb onto the membrane surface, suggesting a potentially distinct partitioning mechanism attributed to their structural rigidity. These findings highlight the diverse membrane interactions and partitioning mechanisms exhibited by different structural classes of AMPs.

**Graphical abstract:**

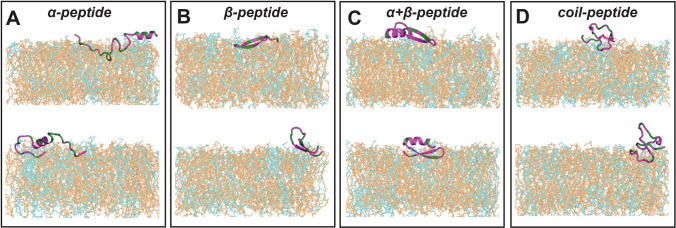

**Supplementary Information:**

The online version contains supplementary material available at 10.1007/s00232-024-00318-z.

## Introduction

Antimicrobial peptides (AMPs) are short chains of amino acids, typically consisting of 12 to 50 amino acids and weighing less than 10 kilodaltons (kDa). These molecules are characterized by their cationic (positively charged) and amphipathic (containing both hydrophobic and hydrophilic regions) nature. Unlike conventional antibiotics agents, AMPs employ alternative mechanisms to inhibit microbial growth (Wang et al. 2016; Tossi et al. 2000; Mcphee and Hancock 2005; Hancock and Sahl 2006; Boucher et al. 2009; Magiorakos et al. 2012; Li et al. 2012). AMPs are present in various organisms across different classes of life and are often found as secondary metabolites in tissues and mucous membranes. Many of these peptides exhibit a broad range of antibacterial activity, enabling them to effectively eliminate diverse microorganisms (Rima et al. 2021; Mahlapuu et al. 2016). Due to their evolutionary conservation in genomes, AMPs are considered essential components of the host defense system against microbial threats (Zasloff 2002a; Hancock 2000; Tossi et al. 2000; Mcphee and Hancock 2005; Hancock and Sahl 2006). AMPs can be categorized based on their source, secondary structure, and amino acid composition (Huan et al. 2020a; Epand and Vogel 1999; Wu et al. 1999). They can be derived from various sources such as plants, amphibians, insects, microorganisms, aquatic organisms, and mammals. The presence of a secondary structure, particularly helices, is long considered to be a crucial characteristic of AMPs. However, there are AMPs that exhibit secondary structures other than helices, including beta strands/sheets, a combination of helices and strands, or even coil structures lacking a distinct secondary structure. Another classification criterion for AMPs is the amino acid composition, which categorizes them as proline-rich, histidine-rich, tryptophan-rich, glycine-rich, or arginine-rich peptides (Huan et al. 2020a). The primary mode of action involves disrupting the integrity of microbial membranes, although they can also exert intracellular functions (Rima et al. 2021).

AMPs have the ability to interact with membranes even at low concentrations in their monomeric state, and these interactions can significantly impact membrane properties. This ranges from thinning of the membrane due to partial partitioning of the peptides to complete translocation into the cellular environment (Huang and Charron 2017; Heller et al. 2000; Mecke et al. 2005; Nicolas 2009; Maria-Neto et al. 2015). However, the precise mechanisms underlying the antimicrobial action of AMPs are not fully understood and are thought to involve several processes, including sensing, partitioning, disruption, and eventual cell lysis. The key components of many AMPs are charged and hydrophobic residues, which have been implicated in the initial sensing and differentiation of bacterial membranes from host membranes, as well as in subsequent partitioning processes (Zasloff 2002b; Mcphee and Hancock 2005; Takahashi et al. 2017; Uppu et al. 2016; Ganewatta and Tang 2015; Yang et al. 2018). However, it has become increasingly clear that this simplistic view may not capture the complete picture.

Recent studies have revealed that AMPs can sense not only differences in lipid components with opposite charges but also intrinsic membrane properties such as curvature as part of their mechanism to differentiate between bacterial and host cell membranes (Schmidt and Wong 2013). For instance based on lipid shape factor, conical lipids (e.g., POPE) induce curvature compared to cylindrical lipids (e.g., POPC), which prefer flat membranes, the former being abundant in bacterial membranes, while the latter in host membranes (Li et al. 2017). This mechanism of curvature sensing is synergistic to inherent presence of packing defects influenced by the types and compositions of lipids (Cui et al. 2011; Vamparys et al. 2013; Vanni et al. 2013, 2014). For example, the presence of conical lipids is known to exhibit higher levels of lipid packing defects compared to that of cylindrical lipids (Vamparys et al. 2013; Vanni et al. 2014; Baul and Vemparala 2017). The packing defects, which result from the stochastic dynamics of lipid molecules at the lipid–water interface of model bacterial membranes, have been recently reported to play significant role in partitioning of antimicrobial polymers with varying functional group compositions (Sikdar et al. 2023). These findings highlight the complexity of membrane recognition by AMPs and suggest that a combination of several factors like amino acid composition, secondary structure, lipid composition, packing defects, and curvature may contribute to their antimicrobial activity. Further research is necessary to elucidate the interplay between these different mechanisms and to fully understand the umbrella of antimicrobial action employed by AMPs.

Over the past few years, several research groups have contributed to the understanding and characterization of these lipid packing defects (Wildermuth et al. 2019; Cui et al. 2011; Vamparys et al. 2013; Tripathy et al. 2020; Sikdar et al. 2021, 2022a, 2023). Different methodologies have been employed, including a solvent accessible surface area (SASA)-based method developed by Voth and colleagues (Cui et al. 2011), as well as a Cartesian grid-based approach used by other researchers to scan the lipid–water interface and identify the solvent-exposed lipid atoms associated with packing defects (Vamparys et al. 2013; Vanni et al. 2013). The latter method has been incorporated into the PackMem software (Gautier et al. 2018), which utilizes an algorithm that divides the $x{-}y$ plane of the lipid bilayer into grids and scans along the *z*-direction from the solvent interface down to a level below the average position of the C$_{2}$ atoms of the glycerol moieties. This scanning process allows the identification of regions with low lipid density, and the packing defect sites are characterized quantitatively based on their occupied area (*A*) and qualitatively labeled as “Deep” or “Shallow” relative to the average level of the C$_{2}$ atoms. While this method primarily focuses on projecting the defect area onto the membrane’s $x{-}y$ plane, Tripathy et al. (2020) introduced a quantitative approach to assess both the depth and area of the defects. They achieved this by scanning the local free volume surrounding each lipid atom to identify the defect pockets within the membrane.

The primary objective of this paper is to uncover the underlying principles governing the initial interaction between different structural classes of antimicrobial peptides (AMPs) and bacterial membranes, with a specific focus on lipid packing defects. To achieve this goal, the following approach is undertaken:

1. *Database analysis* an existing database of AMPs is utilized to identify the sequences for which structural information is available. This subset of AMPs is then classified into different structural classes based on specific variables or criteria.
2. *Selection of representative AMPs* from the identified structural classes, representative AMPs are chosen to ensure a diverse representation of structural characteristics.
3. *Molecular dynamics simulations* molecular dynamics simulations are conducted for the selected representative AMPs in the presence of model bacterial membranes. These simulations provide atomistic insights into the dynamic behavior and early stages of interaction of AMPs that involve recognition and subsequent partitioning mechanisms into membranes.

## Results

### Identification of Structural Classes

A dataset containing 4039 antibacterial sequences is obtained from the DRAMP database (Shi et al. 2021; Kang et al. 2019). Each sequence in the dataset is associated with various attributes such as name, SWISS-PROT entry, family, gene, source, activity, protein existence, structure, structure description, PDB ID, Binding Target, PubMed ID, references, and so on. From this dataset, we identify 230 sequences for which 3D structural data are available in the Protein Data Bank (Berman et al. 2000). However, some sequences are derived from larger proteins, and the corresponding PDB structures contain the entire protein rather than just the antimicrobial peptide (AMP). To rectify this issue, we manually examine the dataset and select a set of 178 sequences that have 3D structures specifically for the peptides, excluding the larger protein structures.

Properties such as hydrophobicity, net charge, and amphipathicity are crucial for AMPs (Mishra and Wang 2012; Huan et al. 2020b), and these properties are calculated based on the amino acid side chains of the selected sequences. For each sequence, the counts of each property are obtained by classifying the amino acid side chains. To assess the contribution of each property in a given sequence, the counts are normalized based on the sequence length. The contribution estimates the significance of each property in the sequence. Firstly, a histogram is generated to visualize the distribution of sequence lengths, with a bin width of 5 (Supplementary Information, Fig. S.1). The analysis reveals that the sequence lengths of AMPs range from a minimum of 9 to a maximum of 93. While the range is broad, the majority of peptides have lengths between 10 and 45, indicating that AMPs commonly fall within this range. Box plots are then used to depict the percentage contribution of sequence properties (Supplementary Information, Fig. S.2). The results indicate that, on average, AMP sequences consist of approximately 40 to 60% non-polar residues, 10 to 40% polar residues, 15 to 30% positively charged residues, and up to 10% negatively charged residues. These box plots provide valuable insights into the amino acid composition of AMPs, highlighting the need for a balanced distribution of different types of residues. Achieving the right balance is crucial as it directly influences the physicochemical properties of the peptides, which, in turn, play a crucial role in their antimicrobial activity.

In addition to sequence-level properties, the secondary structure (SS) content of AMPs plays a vital role in their mechanism of action (Zhang et al. 2016). The secondary structure elements, such as helix, sheet, coil, and turn, are determined using the STRIDE algorithm (Frishman and Argos 1995) implemented in Visual Molecular Dynamics (VMD; Humphrey et al. 1996). Similar to the physicochemical properties, the secondary structural contents are normalized with respect to the sequence length to enable comparative analysis. The distribution of secondary structural elements, such as helix, strand, and coil, within the selected AMP dataset, is shown in Fig. S.3. The analysis reveals that AMPs are designed with a preference for the helical structure over others. To gain insight into the relationship between structure and sequence properties, the SS contents for each of the selected AMPs are plotted against the respective sequence lengths to generate scatter plots corresponding to helix, sheet, and coil contributions, as shown in Fig. S.3B–D. It is evident that short AMPs with a sequence length of around 20 amino acids are predominantly helical, whereas with increasing peptide length (40 amino acids), the decrease in helix content is compensated by sheet content. It is also worth noting that out of the 178 structures in the filtered dataset, 8 of them are completely coil in nature. The list of the PDBs and their secondary structure content can be seen in Fig. S.4 (Supplementary Information).

**Fig. 1:**
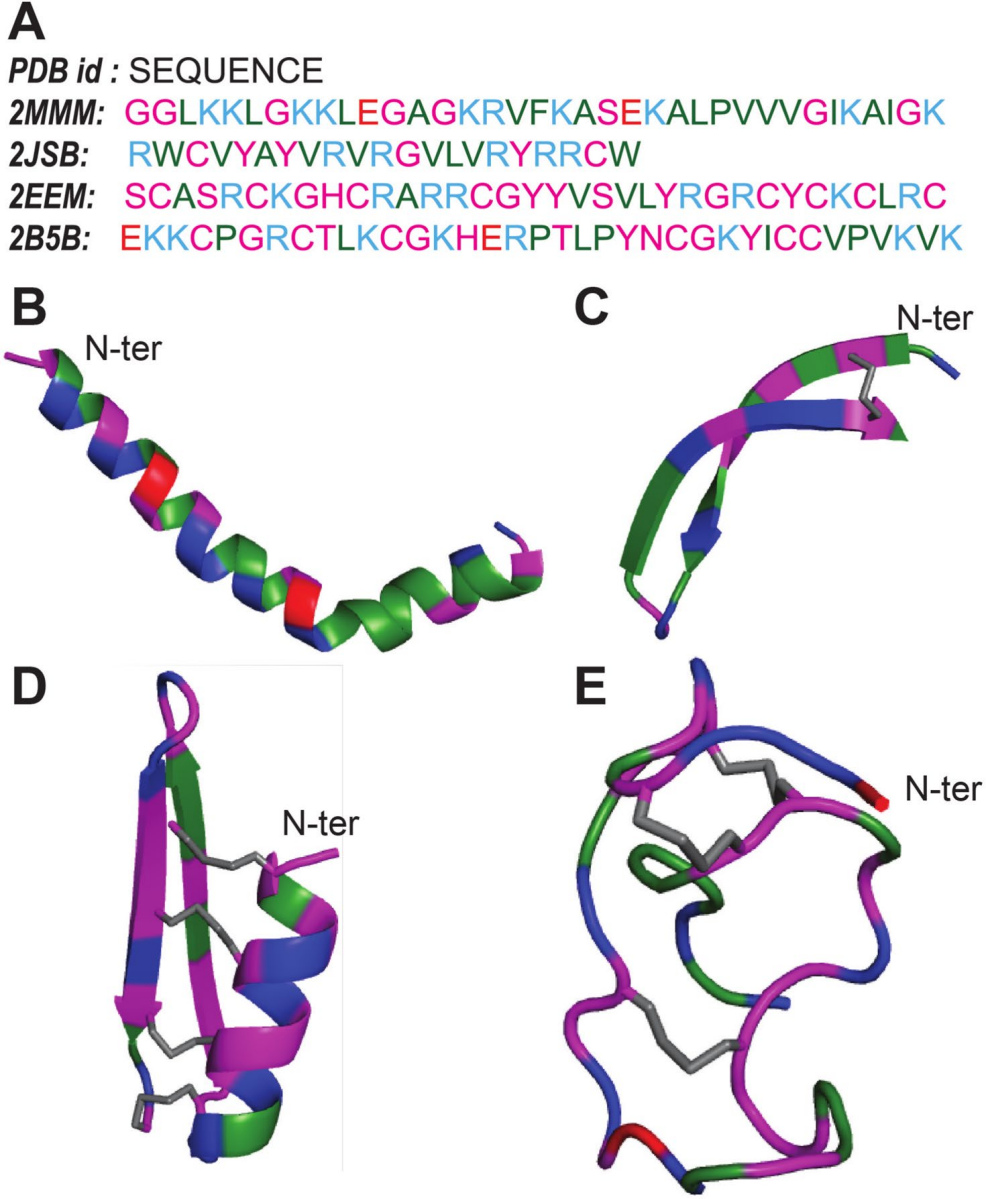
**A** Amino acid sequence and **B**–**E** PDB structures for the representative AMPs belonging to different structural classes considered in the present study. The hydrophobic (green), polar (magenta), basic (blue), acidic (red) residues and disulfide bonds (gray) are shown

Based on this analysis of physicochemical and structural properties, we have identified four representative AMPs as indicated with an arrow in Fig. S.3 that had the highest secondary structural propensity in their respective classes (Mishra and Wang 2012) , which include $\upalpha$-helix, $\upbeta$-sheet, $\upalpha$-helix + $\upbeta$-sheet, and disordered/coil structures (see Supplementary Information, Fig. S.4). Note that, for the $\upalpha$-helix + $\upbeta$-sheet representative, both secondary structures contribute equally. The AMPs chosen as representatives are $\upalpha$-helical Aedesin (*PDB ID 2MMM*) (Godreuil et al. 2014); Arenicin-1 (*PDB ID 2JSB*), with a single disulfide bond-constrained $\upbeta$-sheet conformation (Andrä et al. 2008); Mytilin (*PDB ID 2EEM*), an $\upalpha$-helix + $\upbeta$-sheet structure with four disulfide bonds (Roch et al. 2008); and a disordered/coil Tewp (*PDB ID 2B5B*) peptide stabilized by the presence of three disulfide linkages (Chattopadhyay et al. 2006). These peptides will be referred to as $\upalpha$-peptide, $\upbeta$-peptide, $\upalpha +\upbeta$-peptide, and coil-peptide, respectively; the sequence, structure, and the corresponding disulfide bond networks are illustrated in Fig. 1. These peptide conformations have been determined from solution NMR experiments. The presence of one or multiple disulfide linkages in the chosen AMPs ensures that structure determination is robust, irrespective of solvent or membrane mimicking conditions. Furthermore, the abundance of cysteine residues and thereby the prevalence of disulfide linkages within AMPs, especially those belonging to $\upbeta$-peptide, $\upalpha +\upbeta$-peptide, and coil-peptide, are well documented in earlier studies and hence represent general features of different structural classes (Hammami et al. 2009; Tam et al. 2015; Liu et al. 2010; Koehbach and Craik 2019).

To investigate the effects of secondary structure on the partitioning mechanisms of the representative AMPs, all-atom molecular dynamics (MD) simulations are performed in the presence of a model bacterial membrane composed of POPE–PG lipids (see Methods Section for details). We run two sets of simulations, indicated as Replica 1 and Replica 2, for each AMP–membrane systems by choosing different initial orientation and / or conformation of these peptides. It is important to emphasize that although membrane-active peptides may undergo secondary structure transitions upon partitioning from the solution to the membrane phase, the motivation of the present study is to determine the initial events of membrane recognition and the differential partitioning mechanisms employed by “*experimentally pre-defined*” different secondary structural representatives of AMPs in light of lipid packing defect sensing. The pipeline of the procedure to set up the MD simulations is shown in Fig. S.5.

### Partitioning of AMPs into Model Membranes

**Fig. 2:**
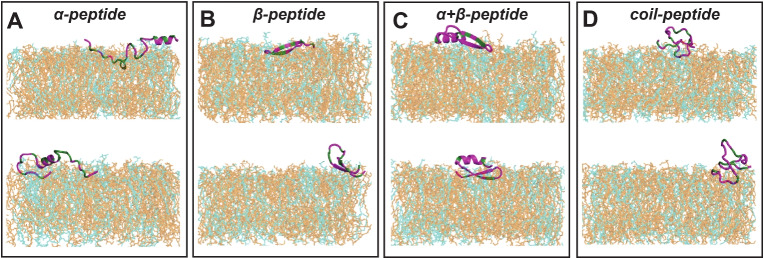
The final snapshots indicating diverse binding modes of the four AMP–membrane systems after the end of both sets of simulations : Replica 1 (top panel) and Replica 2 (bottom panel), where the hydrophobic and hydrophilic residues of AMPs are colored in green and magenta, respectively. The POPE and POPG lipid molecules in the membrane are colored as orange and cyan, respectively. Water and ions are not shown for clarity

In this section, we aim to quantify the interactions between the representative AMPs and the model bacterial membrane. The initial setup of AMPs near the model membrane corresponding to Replica 1 and Replica 2 simulations can be seen in Fig. S.6. and Fig. 2 displays snapshots of the peptides and membrane at the end of both sets of simulation runs. Herein, we present detailed analysis of *Replica 1* simulations and later compare with the second set of simulations. All four peptides exhibit varying degrees of initial partitioning into the model bacterial membranes, with the $\upalpha$-peptide displaying the deepest insertion within the simulation, visually at the end of simulation time scale considered in this study. To assess the structural changes of the peptides, we measure the root mean squared deviation (RMSD) and radius of gyration ($R_{\text {g}}$), which are depicted in Fig. S.7. The $\upalpha$-peptide exhibits the greatest deviation from the initial crystal structure during its interaction with the membrane. These structural changes are further reflected in the variations of $R_{\text {g}}$ and the evolution of secondary structure of the peptides, as illustrated in Fig. S.8. Specifically, for the $\upalpha$-peptide, as the simulation progresses and the peptide–membrane interaction intensifies, a noticeable loss of $\upalpha$-helical content is observed (Fig. S.8A). However, only moderate fluctuations in secondary structure contents are observed for $\upbeta$-, $\upalpha +\upbeta$- and coil-peptides, owing to structural stability provided by the presence of one or multiple disulfide bonds. These results highlight the correlation between the structural stability of the AMPs and insertion mechanism via AMP structural deformation and may underlie different partitioning mechanisms proposed in the literature (Shai 2002; He and Lazaridis 2013; Nguyen et al. 2011; Jenssen et al. 2006; Yeaman and Yount 2003).

**Fig. 3:**
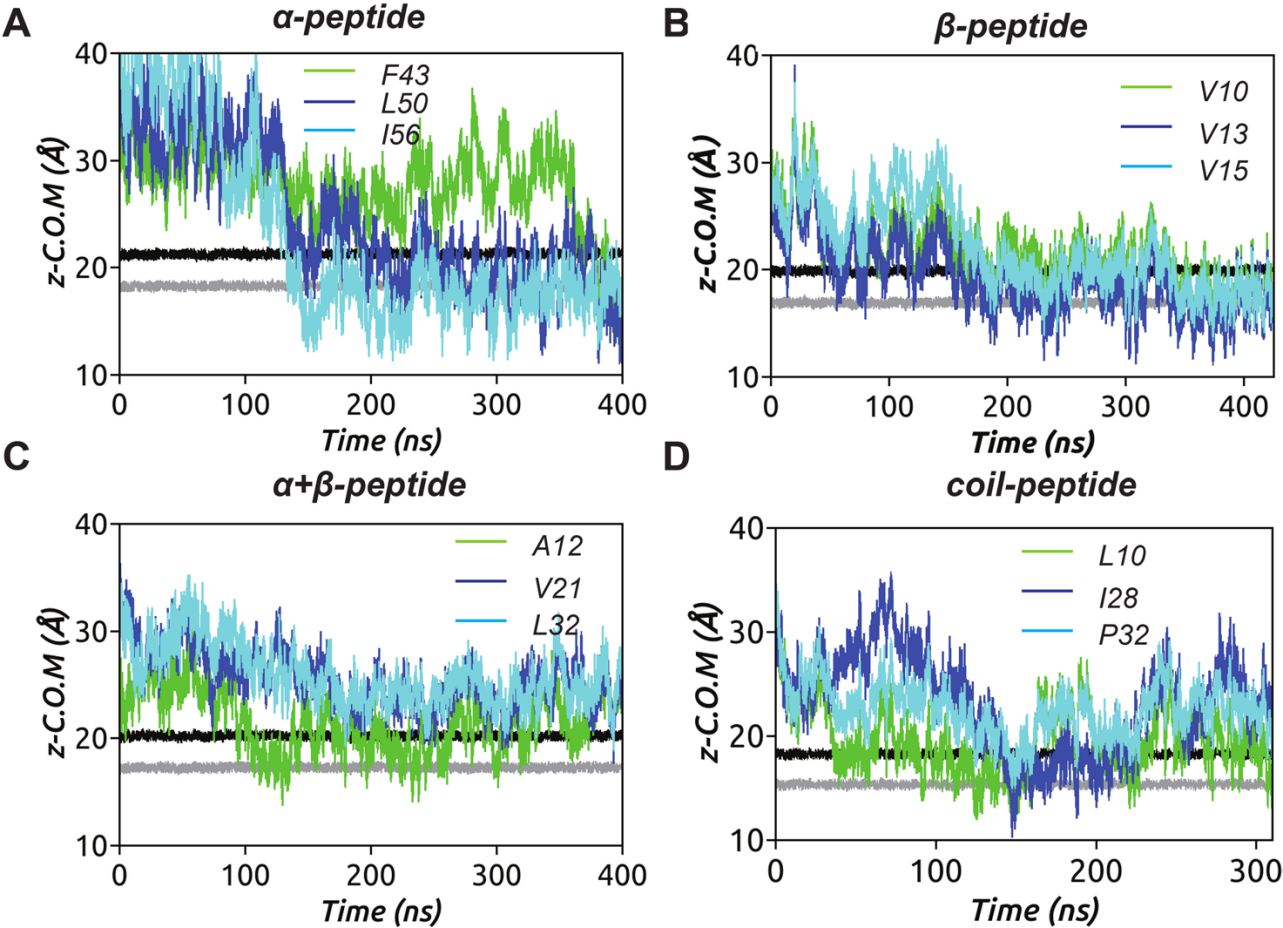
Partitioning of key hydrophobic residues into the model bacterial membrane during the simulation for four AMPs considered (Replica 1). The locations of lipid headgroup P atoms (black) and C$_{2}$ atoms (gray) of glycerol moieties of the upper leaflet, along the membrane normal, of the model bacterial membrane are also shown

**Fig. 4:**
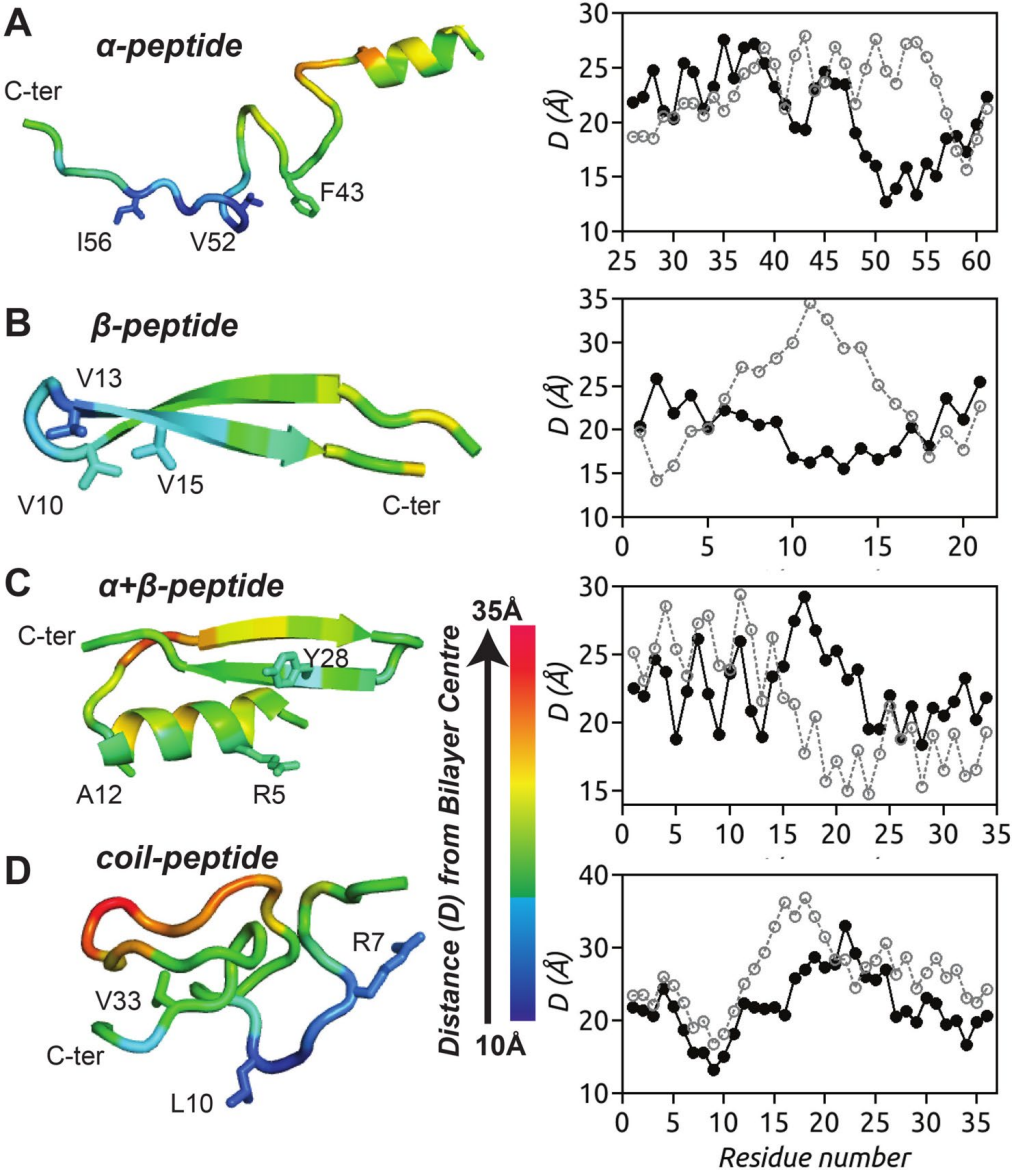
The average residue insertion depths of the four AMPs are calculated as distance from bilayer center averaged over last 50 ns of Replica 1 simulation runs. The color scale indicates, the smaller the value, the deeper is the insertion (left panel). A comparison of average insertion depths calculated from Replica 1 (black, solid circle) and Replica 2 (gray, open circle) sets of simulations as a function of residue number is shown (right panel)

To gain further insights into the dynamics of peptide insertion into the membrane environment, we tracked the time evolution of the *z*-coordinate trajectories of key hydrophobic residues in each peptide, as depicted in Fig. 3 and hydrophilic residues in Fig. S.9. Membrane recognition via electrostatic interaction is initiated by the positively charged C-terminal lysines of the $\upalpha$-peptide (see Fig. S.9), followed by coordinated hydrophobic residue insertions starting from C-terminal toward N-terminal (Fig. 3A). A similar trend is observed for the $\upbeta$-peptide insertion, where cationic arginine R11 from the tip of it’s hairpin motif initiates primary contact with the model membrane (see Fig. S.9), followed by flanking hydrophobic residue insertions in a coordinated manner as shown in Fig. 3B, resulting in a tilted binding mode (see Fig. 2B). In contrast, the residue insertions of the $\upalpha +\upbeta$-peptide and coil-peptide are not deep and represent a surface-adsorbed state (see Fig. 2C, D). This observation is attributed to their structural rigidity imposed by the presence of multiple (four and three, respectively) disulfide bonds, whereas the absence or presence of a single disulfide bond as in the $\upalpha$- and the $\upbeta$-peptide, respectively, allows for conformational malleability, enabling deeper partitioning of residues. These observations are further confirmed by calculating the average insertion depths of individual residues as the distance from bilayer center and mapped on to the respective AMPs (Fig. 4). As can be seen, the $\upalpha$-peptide unfolds at the C-terminal and a large unstructured part of the $\upalpha$-peptide inserts deep into the membrane. Although the coil-peptide remains largely surface adsorbed, a number of residues seem close to the bilayer center compared to the other AMPs, but fewer than the $\upalpha$-peptide. It is to be noted that the coil-peptide considered in this study also has disulfide bonds holding the structure together.

**Fig. 5:**
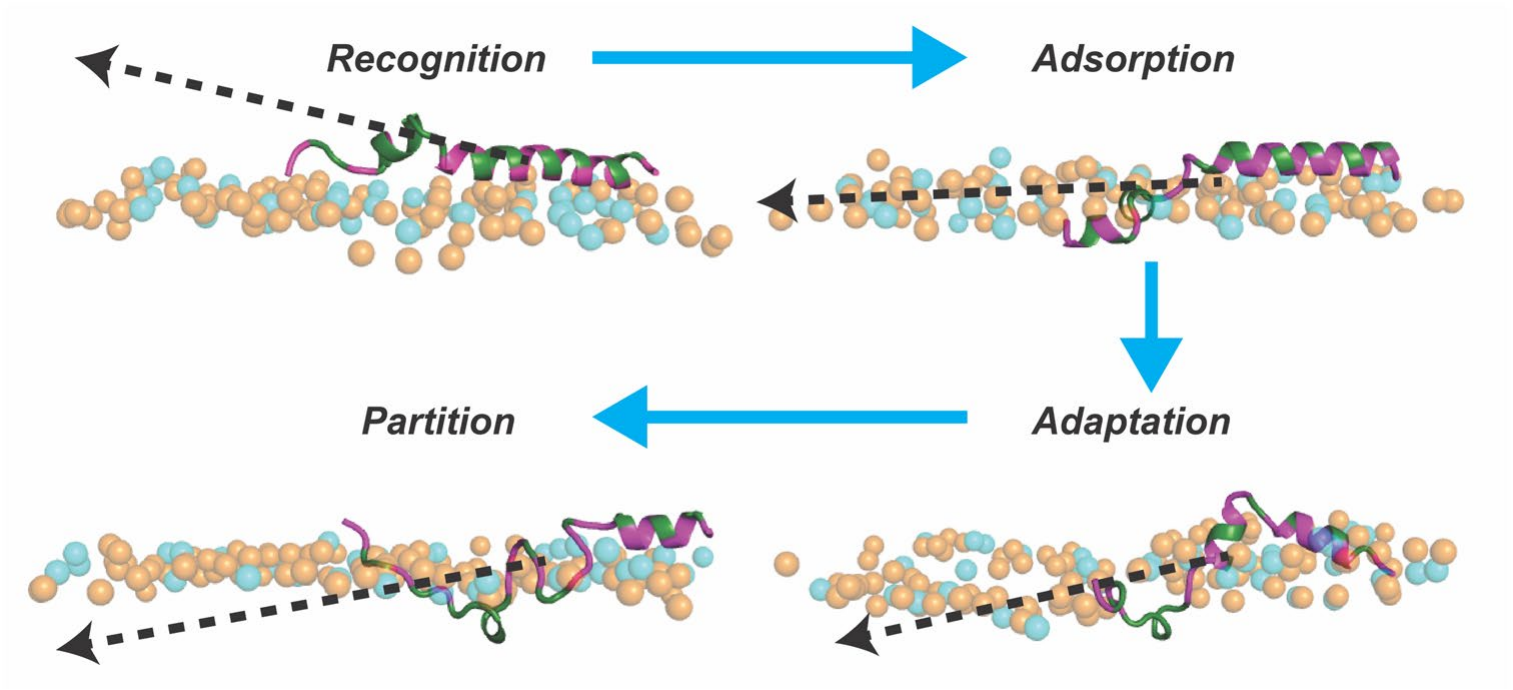
The different stages of $\upalpha$-peptide interacting with a model bacterial membrane composed of POPE (orange) and POPG (cyan) lipids are illustrated. The peptide–membrane recognition is driven by electrostatic interactions between hydrophilic (magenta) peptide residues and lipid headgroups, followed by adsorption on the membrane surface. The unfolding of $\upalpha$-peptide favors conformational adaptation such that hydrophobic (green) residues face membrane interior, while the hydrophilic ones reside close to lipid headgroups, subsequently gaining facial amphiphilicity, resulting in peptide partitioning. The change in orientation of the 3D-Hydrophobic Moment (3D-HM) vector (black dotted arrow) from initially being away from the membrane surface to finally facing the membrane interior demonstrates how $\upalpha$-peptide unfolding facilitates access of hydrophobic residues to the membrane core

These observations strongly suggest that the lack of secondary structure is not the only prerequisite for deep insertion into the membrane. The loss of secondary structure in the initial partitioning scheme has to be followed by structural malleability (for example, in terms of the lack of disulfide bonds) for the most efficient membrane permeability. The observation of deep membrane partitioning for an example $\upalpha$-peptide considered in this study aligns with other studies, where it has been postulated that AMPs with $\upalpha$-helices most likely follow barrel-stave/toroidal mechanisms rather than the surface-dominated carpet mechanism (He and Lazaridis 2013; Kabelka and Vácha 2021; Sato and Feix 2006; Tossi et al. 2000). Based on our simulations, we propose a generic mode of membrane partitioning mechanism for $\upalpha$-helical peptides with structural flexibility, as shown in Fig. 5. The first stage is the recognition stage, involving both electrostatic and sensing lipid packing defects (more on this in the next section) by the $\upalpha$-peptides near the bacterial membranes. This is followed by adsorption and adaptation stages, where the initial unraveling of the flexible elements of the $\upalpha$-peptides can insert themselves in the early stages of partition. We have also calculated the hydrophobic moment vector of the peptide, and the eventual direction of such a vector points to the interior of membranes, suggesting the optimal sequestering of the hydrophobic residues inside the hydrophobic core of the membrane.

**Fig. 6:**
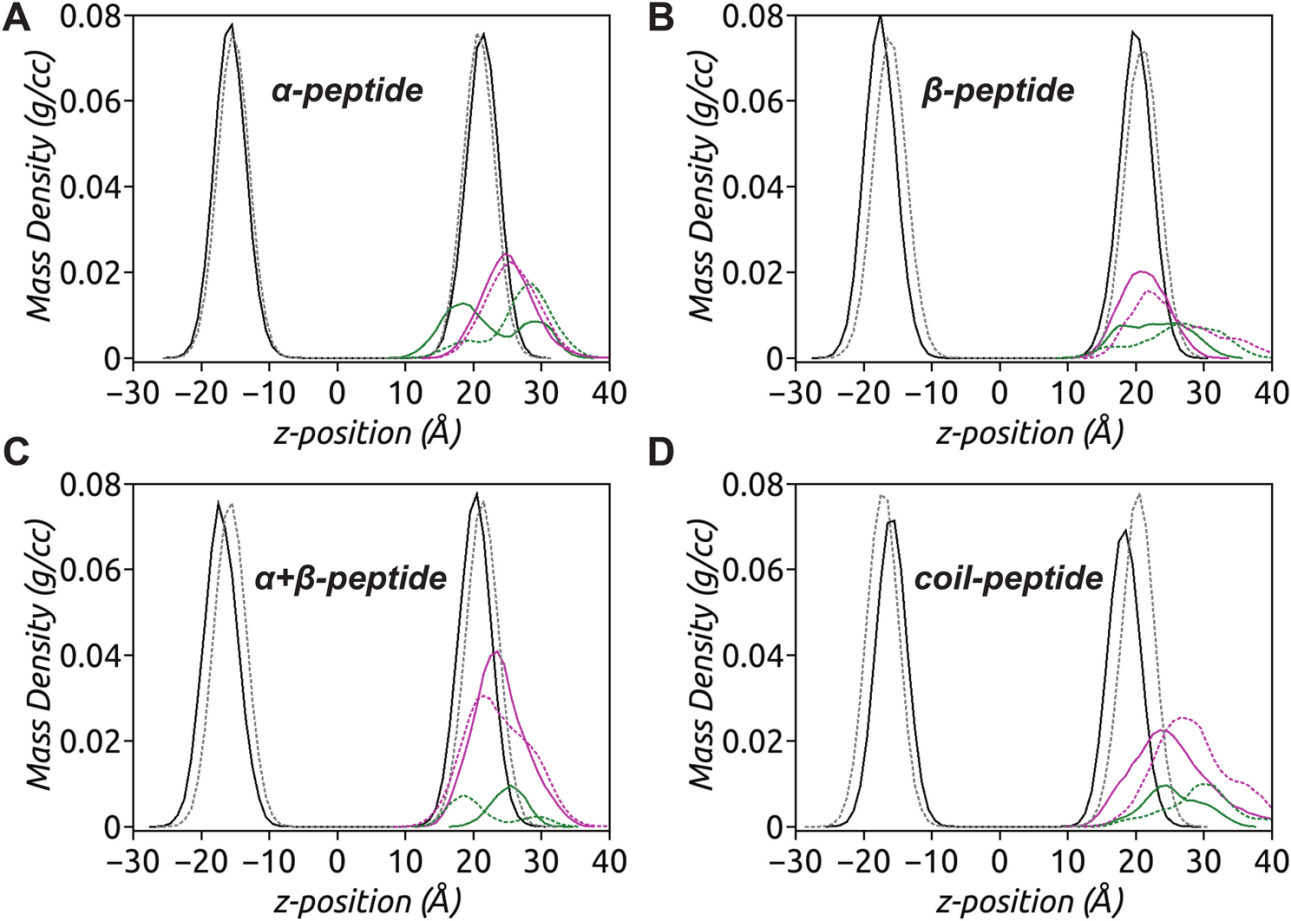
The *z*-density profiles of the four AMPs interacting with model bacterial membrane indicate the location and extent of hydrophobic (green) and hydrophilic (magenta) density components of the peptide with respect to lipid headgroup phosphate atom (P) density (black/gray), corresponding to Replica 1 (solid line) and Replica 2 (dashed line) sets of simulations, calculated over the last 100 ns of respective MD trajectories

To analyze the emergence of amphiphilicity in the partitioned peptides, *z*-density profiles of the four peptides are calculated over the last 100 ns of the simulation. The density profiles, shown in Fig. 6, provide insights into the distribution of hydrophobic and hydrophilic residues in the peptides within the membrane. By comparing the peak positions of the hydrophobic and hydrophilic density profiles, we can estimate the amphiphilicity content of the peptides. The data indicate that the $\upalpha$-peptide exhibits the highest level of amphiphilic character. The location of the hydrophobic residue density peak, further inside the membrane, illustrates the extent of hydrophobic residue partitioning in the $\upalpha$-peptide, aligning with the orientation of the hydrophobic moment vector seen in Fig. 5 (Reißer et al. 2014). After the initial recognition, the $\upalpha$-peptide adheres to the membrane surface and adapts an amphiphilic character through helix unfolding. As a result of this adaptation, the partitioning of one or more hydrophobic residues facilitates the insertion of other hydrophobic residues. The insertion of hydrophobic residues into the membrane can also have profound effects on lipid packing defects that are sensed and exploited by membrane-active peptides. These effects will be further explored in the subsequent section.

Next, we check the reproducibility of our results by comparing Replica 1 and Replica 2 sets of simulations (see Figs. 2, 4, 6). The $\upalpha$-peptide invariably undergoes unfolding in Replica 2 simulations. The initial electrostatic recognition results in the entry of hydrophobic residues from both terminal ends, as indicated by the average insertion depth profile. Although there exists a segregation of hydrophobic and hydrophilic densities in Replica 2 simulations, the acquired amphiphilic character is subdued compared to Replica 1, as the hydrophobic residue stretch, A49 to I56, resides at the lipid headgroup–water interface instead of the membrane interior within our simulation time scale. This may be attributed to the stochasticity in the partitioning dynamics of amphiphilic peptides into the membrane interior. On the other hand, the $\upbeta$-peptide exhibits a different binding mode, albeit tilted, representing another surface-adsorbed state involving its N- and C-terminal arginines, while the tip of the hairpin motif remains solvent-exposed close to the lipid headgroups. The Replica 2 simulation of the $\upalpha +\upbeta$-peptide, based on the calculated average residue depths, indicates a more prominent surface adsorption phenomenon as the $\upbeta$-strands (residues C$_{15}$ to C$_{34}$) remain embedded within the lipid headgroup region compared to that in the Replica 1 simulation. This leads to dragging the hydrophobic density profile closer toward the bilayer center. In contrast, surface adsorption of the coil-peptide is less pronounced in our second set of simulations compared to Replica 1, as evident from the insertion depth and *z*-density profiles, though the trends of both profiles remain conserved. Overall, the results from both sets of simulations are in qualitative agreement, as the surface-adsorbed state of AMPs often involves diverse binding modes characterized by varying initial electrostatic recognition schemes.

### Distribution of Defect Size

**Fig. 7:**
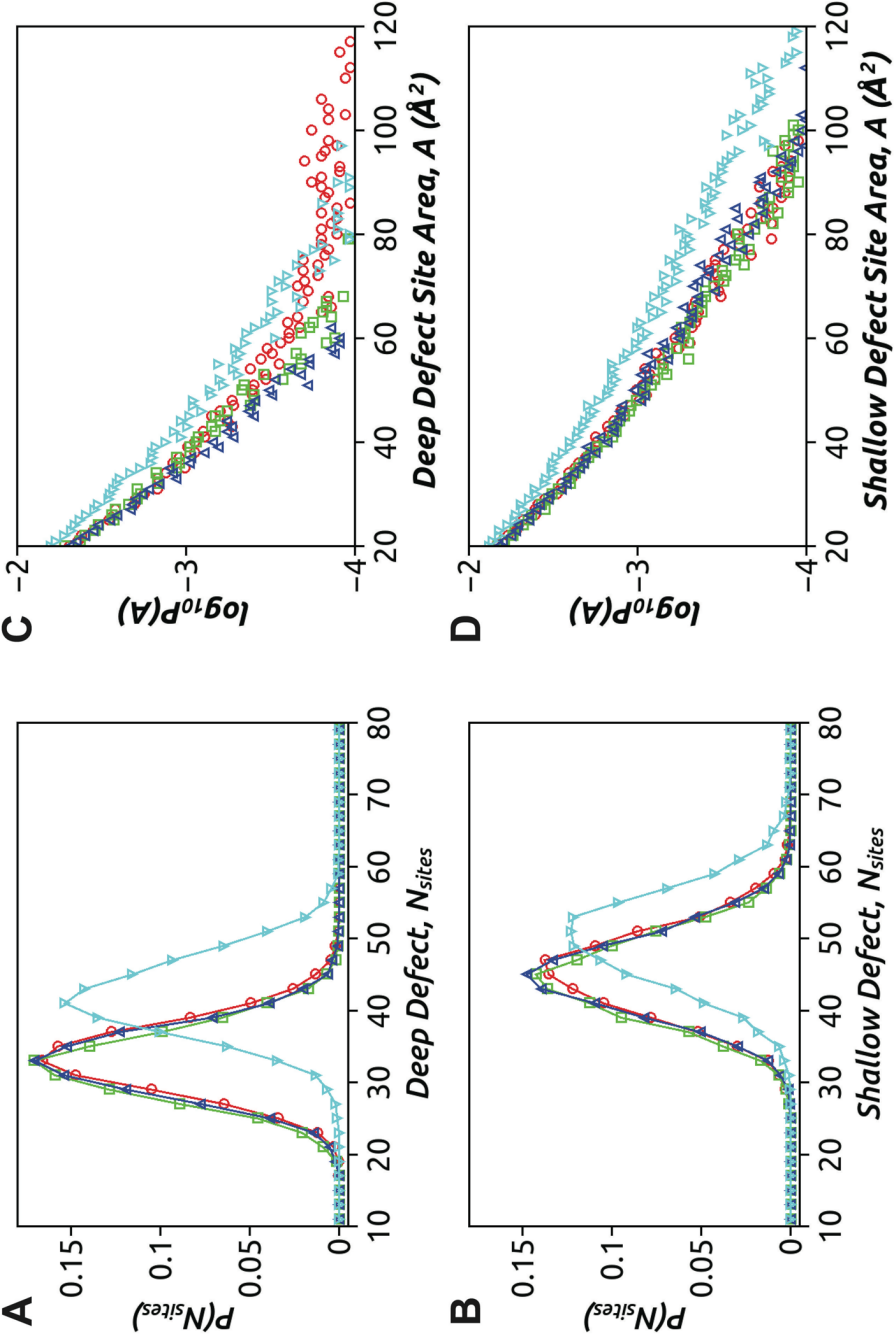
The distribution of number of defect sites per leaflet in a given frame, $P({\text {Nsites}})$, for **A** deep and **B** shallow defects (Replica 1). The size distribution of defect sites, $\log _{10} P(A)$, for **C** deep and **D** shallow defects. The analysis is performed over the last 200 ns trajectories of each AMP–membrane system:$\upalpha$-peptide (ellipse, red), $\upbeta$-peptide (square, green), $\upalpha +\upbeta$-peptide (up-triangle, blue) and coil-peptide (down-triangle, cyan)

In this section, we investigate how different classes of AMPs modulate and influence lipid packing defects in model bacterial membranes. The analysis of lipid packing defects is conducted using the PackMem software (Gautier et al. 2018) over the last 200 ns of each system. To characterize the abundance of defect sites per leaflet in a given frame, denoted as ${\text {Nsites}}$, we examine the distributions $P({\text {Nsites}})$ separately for deep and shallow defects. The results are presented in Fig. 7A and B, respectively. The average number of deep defect sites per leaflet ($\langle {\text {Nsites}}\rangle \sim 32$) is found to be similar for the $\upalpha$-, $\upbeta$-, and $\upalpha +\upbeta$-peptides, as indicated by the overlapping distributions in Fig. 7A. However, the coil-peptide induces a higher abundance of deep defect sites ($\langle {\text {Nsites}}\rangle \sim 42$) compared to the structured AMPs. A similar trend is observed for the distributions of shallow defect sites in Fig. 7B. The coil-peptide also leads to a greater number of shallow defects compared to the structured AMPs. Overall, these results suggest that the coil-peptide, with its disordered nature, engages in larger surface contacts with the bilayer, leading to an increased number of lipid packing defects compared to the structured AMPs.

**Fig. 8:**
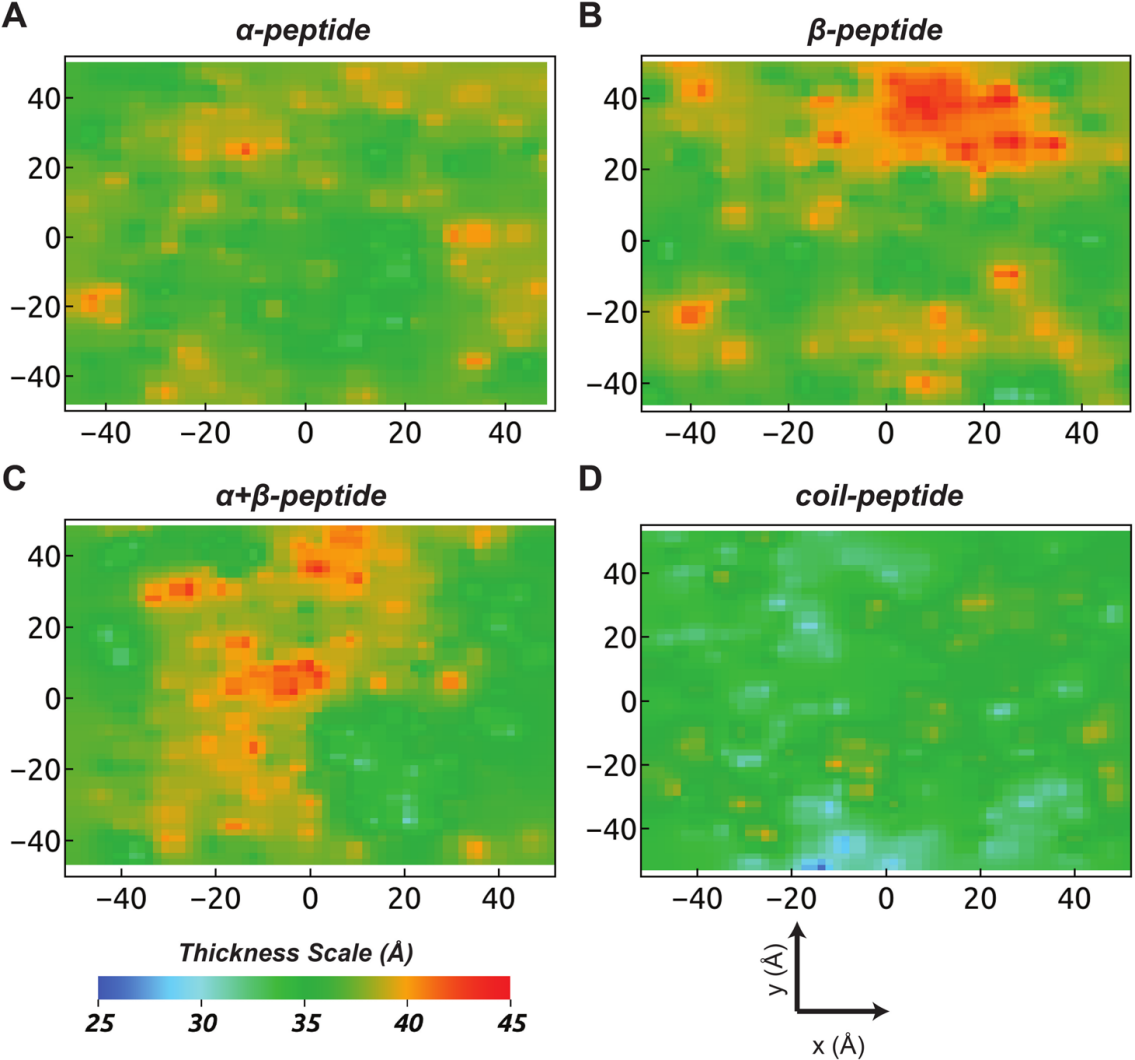
The 2D-thickness maps of model bacterial membrane in the presence of the four AMPs (Replica 1). The thickness maps based on inter-leaflet P–P distance are generated using a $2\times 2\,{ \mathop {\text{A}}\limits^{ \circ }   }^{2}$ resolution along the $x{-}y$ plane for the final MD snapshots of respective trajectories

The size distribution, denoted as *P*(*A*), of defect sites is an important aspect to characterize the effects of structurally diverse AMPs. The defect size distributions, represented in semi-log scale as $\log _{10} P(A)$, are illustrated in Fig. 7C, D. It is interesting to observe that even though the number of deep defect sites is similar across the three structured peptides, the size distributions of deep defects exhibit significant differences (Fig. 7C). The coil-peptide shows a high population of moderate-sized deep defects ($\sim 40 {-} 70\, { \mathop {\text{A}}\limits^{ \circ }   }^{2}$), followed by the $\upalpha$-peptide, $\upbeta$-peptide, and $\upalpha +\upbeta$-peptide. However, larger deep defect sites ($> 80\, { \mathop {\text{A}}\limits^{ \circ }   }^{2}$) are more pronounced in the presence of the $\upalpha$-peptide, particularly due to the insertion of several hydrophobic residues into a single co-localized large deep defect site. The observed pattern in deep defect size distributions can be attributed to the predominance of hydrophobic residues in the $\upalpha$-peptide, while the $\upalpha +\upbeta$-peptide has the least number of such residues. In contrast, the size population of shallow defect sites demonstrates that the coil-peptide not only enhances the number but also the size of shallow defects compared to the structured peptides, which exhibit similar distributions (Fig. 7D). This, in particular, is also evident from membrane response in terms of the corresponding 2D-thickness maps generated for the final snapshots of AMP–membrane systems (Fig. 8) compared to control POPE–PG system without any AMPs (see Fig. S.10). It is clearly evident that owing to the abundance of defect sites in the presence of the coil-peptide, the model bacterial membrane exhibits global thinning with values between $\sim 30$ and $35\,{ \mathop {\text{A}}\limits^{ \circ }   }$. It is because of this enhanced thinning that the residues of the coil-peptide appear closer to bilayer center despite being in a surface-adsorbed state. Membrane thinning is also induced for the $\upalpha$-peptide insertion concomitant with the appearance of defect sites. In contrast, membrane thinning is compromised and only locally induced in the presence of the $\upbeta$-peptide and the $\upalpha +\upbeta$-peptide as compared to the control (Fig. S.10), consistent with their observed degree of partitioning and induced packing defect sites.

**Fig. 9:**
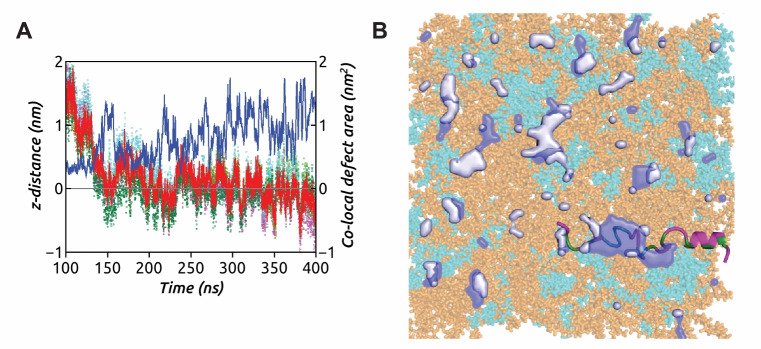
**A** The insertion dynamics of center of mass of hydrophobic residues (red line) from C-terminal of the $\upalpha$-peptide into a co-localized deep defect site, the area fluctuations being shown in blue (Replica 1). Center of masses of individual hydrophobic residues is indicated in dots of different colors. **B** The final snapshot of $\upalpha$-peptide–POPE (orange)/PG (cyan) system illustrates the location and extent of the co-localized deep defect (dark blue) surrounded by shallow defect sites (light blue). The hydrophobic residues of the peptide are shown in green, while the hydrophilic ones in magenta

We now discuss the co-operative interplay involving the appearance of a defect site on the membrane surface co-localized with the $\upalpha$-peptide (Fig. 9). As mentioned earlier, the C-terminal unfolding of the $\upalpha$-peptide precedes the hydrophobic residue insertions. We consider the center of mass of these hydrophobic residues from the unfolded helix and simultaneously track the insertion dynamics using the *z*-distance of this center of mass from the average level of C$_{2}$ atoms in POPE–PG lipids along the membrane normal (*z*-direction). We also monitor the appearance of any underlying packing defects on the membrane surface (Fig. 9A). It is observed that at around 150 ns, a large co-localized deep defect arises, favoring the transient insertion of the unfolded part of the helix, as indicated by negative values of the z-distance. This co-localized deep defect site continues to grow over time, facilitating discrete events of hydrophobic residue insertions and resulting in the partitioning of the $\upalpha$-peptide. This process is characterized by the sensing of lipid packing defects. The final snapshot illustrating the insertion of the $\upalpha$-peptide into this co-localized deep defect site is shown in Fig. 9B. The location of defect sites for the other AMPs is shown in Supplementary Information, Fig. S.10.

## Discussion

The role of lipid packing defects in the recruitment of amphiphilic molecules has been extensively studied in various systems. Hydrophobic nanoparticles, disaccharides, and proteins have been shown to partition into membranes by sensing lipid packing defects (Van Lehn et al. 2014; Moiset et al. 2014; Vanni et al. 2013; Wildermuth et al. 2019; Ouberai et al. 2013; Pinot et al. 2014; Vanni et al. 2014; Garten et al. 2015; Read et al. 2015). The concept of the amphipathic lipid packing sensor (ALPS) motif, commonly found in peripheral membrane proteins, has shed light on the role of hydrophobic residues in sensing and inserting into lipid packing defects (Vanni et al. 2013; Wildermuth et al. 2019; Van Hilten et al. 2020; Vanni et al. 2014). Vanni et al. demonstrated that the hydrophobic residues of the ALPS motif act as lipid packing sensors and subsequently insert into pre-existing packing defects (Vanni et al. 2013). However, a later study suggested that the ALPS motif in close proximity to the membrane can promote the formation of new defects, indicating that the pre-existence of a packing defect is not necessary for peptide insertion (Wildermuth et al. 2019).

Recent research has also highlighted the importance of lipid packing defects in regulating the partitioning mechanism of viral peptides and antimicrobial polymers in membranes. For example, in the case of the Hepatitis A virus 2B (HAV-2B) peptide, the presence of lipid packing defects facilitated peptide partitioning, while a cholesterol-rich bilayer with fewer defects inhibited partitioning (Sikdar et al. 2021, 2022a). Another study demonstrated how antimicrobial polymers with different chemical compositions explored and occupied lipid packing defects at various depths, exhibiting different partitioning mechanisms into model bacterial membranes (Sikdar et al. 2022b). Investigations by Voth and co-workers have revealed that packing defects increase with an increase in membrane curvature (Cui et al. 2011). The synergistic effect of membrane curvature and lipid tail unsaturation on interfacial packing defects has also been confirmed, where increasing lipid unsaturation or introducing conical lipids into flat bilayers led to a defect size distribution resembling that of a positively curved bilayer (Vanni et al. 2014). Furthermore, studies have shown that membrane thinning enhances lipid packing defects. By applying external force in molecular dynamics simulations, Hilten et al. developed a protocol to vary membrane thickness and found a higher probability of finding large defects in thin membranes compared to membranes of normal thickness (Van Hilten et al. 2020).

In this study, a combination of bioinformatics and molecular dynamics simulations is utilized to explore how diverse classes of antimicrobial peptides (AMPs) interact with model membranes and partition within them. The primary objective is to uncover potential correlations between the structural characteristics of AMPs and their mechanisms of membrane partitioning. The simulation results reveal distinct membrane interactions among the various structural classes of AMPs, particularly in relation to the generation and interaction with lipid packing defects. Notably, AMPs with a structure-less coil conformation induce global thinning of bacterial membrane by generating higher number of defect sites, which are larger in size compared to other classes of AMPs. Conversely, AMPs with a helical component tend to acquire an amphiphilic conformation and demonstrate significant insertion into the membrane via inducing and sensing lipid packing defects. On the other hand, beta sheet AMPs suggest a distinct partitioning mechanism due to their structural rigidity imposed by the presence of disulfide bonds. As a result, these beta sheet AMPs tend to adsorb onto the membrane surface via electrostatic interactions and induce fewer packing defects. Although the intrinsic origin of packing defects is governed by lipid type and composition of the bacterial membrane, the presence of different AMPs can modulate them to varying extents, influenced by a co-operative interplay of several factors such as primary sequence, secondary structure, hydrophobic residue insertion, and surface area contacts. This indicates the existence of diverse mechanisms for perturbing bacterial membranes. In the field of designing biomimetic polymers with antimicrobial properties (Palermo et al. 2012; Sikdar et al. 2023; Baul et al. 2014; Baul and Vemparala 2017; Palermo et al. 2013; Kuroda and Caputo 2013), these findings hold significance, as they provide insights into how naturally occurring antimicrobial peptides interact and exploit bacterial membrane lipid packing defects for efficient partitioning. Such knowledge can aid in the better design of antimicrobial polymers. Future studies will also investigate how aggregates of AMPs from different structural classes interact and influence membranes through lipid packing defects, further advancing our understanding of the antimicrobial mechanisms employed by these peptides.

## Models and Methods

A flowchart describing the various steps in the pipeline of data collection, classification of structural classes, and identification of representative AMPs for MD simulations is illustrated in Fig. S.5.

### Analysis of DRAMP Database

DRAMP (Shi et al. 2021; Kang et al. 2019) (Data Repository of AntiMicrobial Peptides) is a manually curated open-source database consisting annotations for diverse set of AMPs including their sequences, structures, activities, literature references, and clinical and physiochemical information. Currently, the database harbors $\sim$ 25,000 entries, of which 6105 are general AMPs (both synthetic and natural), 16,110 patented AMPs and 77 AMPs that are under preclinical trials. Based on the activity of the peptide from the literatures, the database has grouped the compounds into 11 different classes such as antibacterial, antifungal, and so on. This database can be accessed using the specified URL http://dramp.cpu-bioinfor.org/.

### MD Simulation in Model Bacterial Membrane

To model the bacterial membrane, we use a pre-equilibrated membrane patch consisting of 70% POPE (palmitoyl-oleoyl-phosphatidylethanolamine) and 30% POPG (palmitoyl-oleoyl-phosphatidylglycerol) lipids. This membrane composition closely resembles the inner membrane of bacteria (Polyansky et al. 2010). The membrane patch, containing 128 lipids per leaflet, is prepared using the Membrane Builder module of CHARMM-GUI (Jo et al. 2009), which has been utilized in our previous studies on antimicrobial polymers (Baul et al. 2014; Baul and Vemparala 2017; Rani et al. 2021). For each antimicrobial peptide (AMP), we select a random conformation from the solution-state NMR-derived structural ensemble. The protonation states of the peptide residues are determined at neutral pH using PROPKA3 (Søndergaard et al. 2011; Olsson et al. 2011), and hydrogen atoms are added accordingly. To set up the four AMP–membrane systems, the peptides are placed near the upper leaflet of the pre-equilibrated POPE–PG bilayer, ensuring there are no steric clashes. We add sufficient water molecules and ions to maintain a salt concentration of 0.15 M. The all-atom molecular dynamics (MD) simulations of the systems are performed using the TIP3P water model (Jorgensen et al. 1983), along with CHARMM36m (Huang et al. 2017) and CHARMM36 (Klauda et al. 2010) force field parameters for peptide and lipid molecules, respectively. The simulations are conducted using NAMD2.10 (Phillips et al. 2005). Prior to the production run, all systems undergo an energy minimization of 10,000 steps. Then, an equilibration protocol is applied, where positional restraints on peptide heavy atoms are gradually decreased over a period of 3 ns to ensure relaxed starting configurations of the AMP–membrane systems. The simulations are performed under periodic boundary conditions in the isothermal–isobaric ensemble at 1 atm pressure and 310 K, with a time step of 2 fs. The van der Waals interactions beyond 12Å are smoothly truncated using a force-based switching function, while electrostatic interactions are calculated using the Particle mesh Ewald fast Fourier transform. The production runs are carried out for over 300 ns, and subsequent analysis of the equilibrated trajectories is performed using VMD (Humphrey et al. 1996), MEMBPLUGIN (Guixà-González et al. 2014), and PackMem (Gautier et al. 2018). The MD system details can be found in Tables S1 and  S2 .

## Supplementary Information

Below is the link to the electronic supplementary material.

Supplementary file 1 (pdf 22059 KB)

## Data Availability

No datasets were generated or analyzed during the current study.

## References

[CR1] Andrä J, Jakovkin I, Grötzinger J, Hecht O, Krasnosdembskaya AD, Goldmann T, Gutsmann T, Leippe M (2008) Structure and mode of action of the antimicrobial peptide arenicin. Biochem J 410(1):113–122. 10.1042/BJ20071051 17935487 10.1042/BJ20071051

[CR3] Baul U, Vemparala S (2017) Influence of lipid composition of model membranes on methacrylate antimicrobial polymer–membrane interactions. Soft Matter 13:7665–7676. 10.1039/C7SM01211J 28991313 10.1039/c7sm01211j

[CR2] Baul U, Kuroda K, Vemparala S (2014) Interaction of multiple biomimetic antimicrobial polymers with model bacterial membranes. J Chem Phys 141(8):084902. 10.1063/1.4893440 25173040 10.1063/1.4893440

[CR4] Berman HM, Westbrook J, Feng Z, Gilliland G, Bhat TN, Weissig H, Shindyalov IN, Bourne PE (2000) The protein data bank. Nucleic Acids Res 28(1):235–242 10592235 10.1093/nar/28.1.235 PMC102472

[CR5] Boucher HW, Talbot GH, Bradley JS, Edwards JE, Gilbert D, Rice LB, Scheld M, Spellberg B, Bartlett J (2009) Bad bugs, no drugs: no ESKAPE! An update from the Infectious Diseases Society of America. Clin Infect Dis 48(1):1–12. 10.1086/595011 19035777 10.1086/595011

[CR6] Chattopadhyay S, Sinha NK, Banerjee S, Roy D, Chattopadhyay D, Roy S (2006) Small cationic protein from a marine turtle has beta-defensin-like fold and antibacterial and antiviral activity. Proteins Struct Funct Bioinform 64(2):524–531. 10.1002/prot.20963 10.1002/prot.20963 16700051

[CR7] Cui H, Lyman E, Voth GA (2011) Mechanism of membrane curvature sensing by amphipathic helix containing proteins. Biophys J 100(5):1271–1279 21354400 10.1016/j.bpj.2011.01.036 PMC3043213

[CR8] Epand RM, Vogel HJ (1999) Diversity of antimicrobial peptides and their mechanisms of action. Biochim Biophys Acta Biomembr 1462(1–2):11–28 10.1016/s0005-2736(99)00198-4 10590300

[CR9] Frishman D, Argos P (1995) Knowledge-based protein secondary structure assignment. Proteins Struct Funct Bioinform 23(4):566–579. 10.1002/prot.340230412 10.1002/prot.340230412 8749853

[CR10] Ganewatta MS, Tang C (2015) Controlling macromolecular structures towards effective antimicrobial polymers. Polymer (Korea) 63:1–29. 10.1016/j.polymer.2015.03.007

[CR11] Garten M, Prevost C, Cadart C, Gautier R, Bousset L, Melki R, Bassereau P, Vanni S (2015) Methyl-branched lipids promote the membrane adsorption of -synuclein by enhancing shallow lipid-packing defects. Phys Chem Chem Phys 17(24):15589–15597 25824255 10.1039/c5cp00244c

[CR12] Gautier R, Bacle A, Tiberti ML, Fuchs PF, Vanni S, Antonny B (2018) PackMem: a versatile tool to compute and visualize interfacial packing defects in lipid bilayers. Biophys J 115(3):436–444. 10.1016/j.bpj.2018.06.025 30055754 10.1016/j.bpj.2018.06.025 PMC6084522

[CR13] Godreuil S, Leban N, Padilla A, Hamel R, Luplertlop N, Chauffour A, Vittecoq M, Hoh F, Thomas F, Sougakoff W, Lionne C, Yssel H, Missé D (2014) Aedesin: structure and antimicrobial activity against multidrug resistant bacterial strains. PLoS ONE 9(8):1–9 10.1371/journal.pone.0105441 PMC4146511 25162372

[CR14] Guixà-González R, Rodriguez-Espigares I, Ramírez-Anguita JM, Carrió-Gaspar P, Martinez-Seara H, Giorgino T, Selent J (2014) MEMBPLUGIN: studying membrane complexity in VMD. Bioinformatics 30(10):1478–1480. 10.1093/bioinformatics/btu037 24451625 10.1093/bioinformatics/btu037

[CR15] Hammami R, Ben Hamida J, Vergoten G, Fliss I (2009) PhytAMP: a database dedicated to antimicrobial plant peptides. Nucleic Acids Res 37(Suppl 1):963–968 10.1093/nar/gkn655 PMC2686510 18836196

[CR16] Hancock REW (2000) Cationic antimicrobial peptides: towards clinical applications. Expert Opin Investig Drugs 9(8):1723–1729. 10.1517/13543784.9.8.1723 11060771 10.1517/13543784.9.8.1723

[CR17] Hancock REW, Sahl H-G (2006) Antimicrobial and host-defense peptides as new anti-infective therapeutic strategies. Nat Biotechnol 24(12):1551–1557. 10.1038/nbt1267 17160061 10.1038/nbt1267

[CR18] He Y, Lazaridis T (2013) Activity determinants of helical antimicrobial peptides: a large-scale computational study. PLoS ONE 8(6):66440 10.1371/journal.pone.0066440 PMC3680375 23776672

[CR19] Heller WT, Waring AJ, Lehrer RI, Harroun TA, Weiss TM, Yang L, Huang HW (2000) Membrane thinning effect of the -sheet antimicrobial protegrin. Biochemistry 39(1):139–145 10625488 10.1021/bi991892m

[CR21] Huan Y, Kong Q, Mou H, Yi H (2020a) Antimicrobial peptides: classification, design, application and research progress in multiple fields. Front Microbiol. 10.3389/fmicb.2020.582779 33178164 10.3389/fmicb.2020.582779 PMC7596191

[CR22] Huan Y, Kong Q, Mou H, Yi H (2020b) Antimicrobial peptides: classification, design, application and research progress in multiple fields. Front Microbiol. 10.3389/fmicb.2020.582779 33178164 10.3389/fmicb.2020.582779 PMC7596191

[CR23] Huang HW, Charron NE (2017) Understanding membrane-active antimicrobial peptides. Q Rev Biophys 50:10 10.1017/S0033583517000087 29233222

[CR24] Huang J, Rauscher S, Nawrocki G, Ran T, Feig M, Groot BL, Grubmüller H, MacKerell JAD (2017) CHARMM36m: an improved force field for folded and intrinsically disordered proteins. Nat Methods 14(1):71–73. 10.1038/nmeth.4067 27819658 10.1038/nmeth.4067 PMC5199616

[CR25] Humphrey W, Dalke A, Schulten K (1996) VMD: visual molecular dynamics. J Mol Graph 14(1):33–38. 10.1016/0263-7855(96)00018-5 8744570 10.1016/0263-7855(96)00018-5

[CR26] Jenssen H, Hamill P, Hancock REW (2006) Peptide antimicrobial agents. Clin Microbiol Rev 19(3):491–511. 10.1128/CMR.00056-05 16847082 10.1128/CMR.00056-05 PMC1539102

[CR27] Jo S, Lim JB, Klauda JB, Im W (2009) CHARMM-GUI membrane builder for mixed bilayers and its application to yeast membranes. Biophys J 97(1):50–58. 10.1016/j.bpj.2009.04.013 19580743 10.1016/j.bpj.2009.04.013 PMC2711372

[CR28] Jorgensen WL, Chandrasekhar J, Madura JD, Impey RW, Klein ML (1983) Comparison of simple potential functions for simulating liquid water. J Chem Phys 79(2):926–935

[CR29] Kabelka I, Vácha R (2021) Advances in molecular understanding of -helical membrane-active peptides. Acc Chem Res 54(9):2196–2204 33844916 10.1021/acs.accounts.1c00047

[CR30] Kang X, Dong F, Shi C, Liu S, Sun J, Chen J, Li H, Xu H, Lao X, Zheng H (2019) DRAMP 2.0, an updated data repository of antimicrobial peptides. Sci Data 6(1):148. 10.1038/s41597-019-0154-y 31409791 10.1038/s41597-019-0154-y PMC6692298

[CR31] Klauda JB, Venable RM, Freites JA, O’ Connor JW, Tobias DJ, Mondragon-Ramirez C, Vorobyov I, MacKerell AD, Pastor RW (2010) Update of the CHARMM all-atom additive force field for lipids: validation on six lipid types. J Phys Chem B 114(23):7830–7843 20496934 10.1021/jp101759q PMC2922408

[CR32] Koehbach J, Craik DJ (2019) The vast structural diversity of antimicrobial peptides. Trends Pharmacol Sci 40(7):517–528 31230616 10.1016/j.tips.2019.04.012

[CR33] Kuroda K, Caputo GA (2013) Antimicrobial polymers as synthetic mimics of host-defense peptides. Wiley Interdiscip Rev Nanomed Nanobiotechnol 5(1):49–66. 10.1002/wnan.1199 23076870 10.1002/wnan.1199

[CR36] Li Y, Xiang Q, Zhang Q, Huang Y, Su Z (2012) Overview on the recent study of antimicrobial peptides: origins, functions, relative mechanisms and application. Peptides 37(2):207–215 22800692 10.1016/j.peptides.2012.07.001

[CR35] Li J, Koh J-J, Liu S, Lakshminarayanan R, Verma CS, Beuerman RW (2017) Membrane active antimicrobial peptides: translating mechanistic insights to design. Front Neurosci 11:73 28261050 10.3389/fnins.2017.00073 PMC5306396

[CR37] Liu S, Zhou L, Lakshminarayanan R, Beuerman R (2010) Multivalent antimicrobial peptides as therapeutics: design principles and structural diversities. Int J Pept Res Ther 16:199–213 20835389 10.1007/s10989-010-9230-z PMC2931633

[CR38] Magiorakos A-P, Srinivasan A, Carey RB, Carmeli Y, Falagas ME, Giske CG, Harbarth S, Hindler JF, Kahlmeter G, Olsson-Liljequist B, Paterson DL, Rice LB, Stelling J, Struelens MJ, Vatopoulos A, Weber JT, Monnet DL (2012) Multidrug-resistant, extensively drug-resistant and pandrug-resistant bacteria: an international expert proposal for interim standard definitions for acquired resistance. Clin Microbiol Infect 18(3):268–281. 10.1111/j.1469-0691.2011.03570.x 21793988 10.1111/j.1469-0691.2011.03570.x

[CR39] Mahlapuu M, Håkansson J, Ringstad L, Björn C (2016) Antimicrobial peptides: an emerging category of therapeutic agents. Front Cell Infect Microbiol. 10.3389/fcimb.2016.00194 28083516 10.3389/fcimb.2016.00194 PMC5186781

[CR40] Maria-Neto S, Almeida KC, Macedo MLR, Franco OL (2015) Understanding bacterial resistance to antimicrobial peptides: from the surface to deep inside. Biochim Biophys Acta Biomembr 1848(11):3078–3088 10.1016/j.bbamem.2015.02.017 25724815

[CR41] Mcphee JB, Hancock REW (2005) Function and therapeutic potential of host defence peptides. J Pept Sci 11(11):677–687. 10.1002/psc.704 16103989 10.1002/psc.704

[CR42] Mecke A, Lee D-K, Ramamoorthy A, Orr BG, Holl MMB (2005) Membrane thinning due to antimicrobial peptide binding: an atomic force microscopy study of MSI-78 in lipid bilayers. Biophys J 89(6):4043–4050 16183881 10.1529/biophysj.105.062596 PMC1366969

[CR43] Mishra B, Wang G (2012) The importance of amino acid composition in natural AMPs: an evolutional, structural, and functional perspective. Front Immunol 3:221. 10.3389/fimmu.2012.00221 23060873 10.3389/fimmu.2012.00221 PMC3459185

[CR44] Moiset G, Lopez CA, Bartelds R, Syga L, Rijpkema E, Cukkemane A, Baldus M, Poolman B, Marrink SJ (2014) Disaccharides impact the lateral organization of lipid membranes. J Am Chem Soc 136(46):16167–16175 25316578 10.1021/ja505476c

[CR45] Nguyen LT, Haney EF, Vogel HJ (2011) The expanding scope of antimicrobial peptide structures and their modes of action. Trends Biotechnol 29(9):464–472. 10.1016/j.tibtech.2011.05.001 21680034 10.1016/j.tibtech.2011.05.001

[CR46] Nicolas P (2009) Multifunctional host defense peptides: intracellular-targeting antimicrobial peptides. FEBS J 276(22):6483–6496 19817856 10.1111/j.1742-4658.2009.07359.x

[CR47] Olsson MHM, Søndergaard CR, Rostkowski M, Jensen JH (2011) PROPKA3: consistent treatment of internal and surface residues in empirical pKa predictions. J Chem Theory Comput 7(2):525–537. 10.1021/ct100578z 26596171 10.1021/ct100578z

[CR48] Ouberai MM, Wang J, Swann MJ, Galvagnion C, Guilliams T, Dobson CM, Welland ME (2013) Alpha-synuclein senses lipid packing defects and induces lateral expansion of lipids leading to membrane remodeling. J Biol Chem 288(29):20883–20895. 10.1074/jbc.M113.478297 23740253 10.1074/jbc.M113.478297 PMC3774359

[CR49] Palermo EF, Vemparala S, Kuroda K (2012) Cationic spacer arm design strategy for control of antimicrobial activity and conformation of amphiphilic methacrylate random copolymers. Biomacromolecules 13(5):1632–1641. 10.1021/bm300342u 22475325 10.1021/bm300342u

[CR50] Palermo EF, Vemparala S, Kuroda K (2013) Chapter 20. In: Antimicrobial polymers: molecular design as synthetic mimics of host-defense peptides. American Chemical Society, pp 319–330. 10.1021/bk-2013-1135.ch019

[CR51] Phillips JC, Braun R, Wang W, Gumbart J, Tajkhorshid E, Villa E, Chipot C, Skeel RD, Kale L, Schulten K (2005) Scalable molecular dynamics with NAMD. J Comput Chem 26(16):1781–1802. 10.1002/jcc.20289 16222654 10.1002/jcc.20289 PMC2486339

[CR52] Pinot M, Vanni S, Pagnotta S, Lacas-Gervais S, Payet L-A, Ferreira T, Gautier R, Goud B, Antonny B, Barelli H (2014) Polyunsaturated phospholipids facilitate membrane deformation and fission by endocytic proteins. Science 345(6197):693–697 25104391 10.1126/science.1255288

[CR53] Polyansky AA, Ramaswamy R, Volynsky PE, Sbalzarini IF, Marrink SJ, Efremov RG (2010) Antimicrobial peptides induce growth of phosphatidylglycerol domains in a model bacterial membrane. J Phys Chem Lett 1(20):3108–3111. 10.1021/jz101163e

[CR54] Rani G, Kuroda K, Vemparala S (2021) Towards designing globular antimicrobial peptide mimics: role of polar functional groups in biomimetic ternary antimicrobial polymers. Soft Matter 17:2090–2103. 10.1039/D0SM01896A 33439212 10.1039/d0sm01896a

[CR55] Read J, Clancy EK, Sarker M, Antueno R, Langelaan DN, Parmar HB, Shin K, Rainey JK, Duncan R (2015) Reovirus FAST proteins drive pore formation and syncytiogenesis using a novel helix-loop-helix fusion-inducing lipid packing sensor. PLoS Pathog 11(6):1004962 10.1371/journal.ppat.1004962 PMC4464655 26061049

[CR56] Reißer S, Strandberg E, Steinbrecher T, Ulrich AS (2014) 3D hydrophobic moment vectors as a tool to characterize the surface polarity of amphiphilic peptides. Biophys J 106(11):2385–2394 24896117 10.1016/j.bpj.2014.04.020 PMC4052240

[CR57] Rima M, Rima M, Fajloun Z, Sabatier J-M, Bechinger B, Naas T (2021) Antimicrobial peptides: a potent alternative to antibiotics. Antibiotics (Basel). 10.3390/antibiotics10091095 34572678 10.3390/antibiotics10091095 PMC8466391

[CR58] Roch P, Yang Y, Toubiana M, Aumelas A (2008) NMR structure of mussel mytilin, and antiviral–antibacterial activities of derived synthetic peptides. Dev Comp Immunol 32(3):227–238. 10.1016/j.dci.2007.05.006 17628674 10.1016/j.dci.2007.05.006

[CR59] Sato H, Feix JB (2006) Peptide–membrane interactions and mechanisms of membrane destruction by amphipathic -helical antimicrobial peptides. Biochim Biophys Acta Biomembr 1758(9):1245–1256. 10.1016/j.bbamem.2006.02.021. (**Membrane Biophysics of Antimicrobial Peptides**) 10.1016/j.bbamem.2006.02.021 16697975

[CR60] Schmidt NW, Wong GC (2013) Antimicrobial peptides and induced membrane curvature: geometry, coordination chemistry, and molecular engineering. Curr Opin Solid State Mater Sci 17(4):151–163 24778573 10.1016/j.cossms.2013.09.004 PMC4000235

[CR61] Shai Y (2002) Mode of action of membrane active antimicrobial peptides. Pept Sci 66(4):236–248. 10.1002/bip.10260 10.1002/bip.10260 12491537

[CR62] Shi G, Kang X, Dong F, Liu Y, Zhu N, Hu Y, Xu H, Lao X, Zheng H (2021) DRAMP 3.0: an enhanced comprehensive data repository of antimicrobial peptides. Nucleic Acids Res 50(D1):488–496. 10.1093/nar/gkab651 10.1093/nar/gkab651 PMC8728287 34390348

[CR63] Sikdar S, Banerjee M, Vemparala S (2021) Effect of cholesterol on the membrane partitioning dynamics of hepatitis a virus-2B peptide. Soft Matter 17(34):7963–7977. 10.1039/D1SM01019K 34378608 10.1039/d1sm01019k

[CR64] Sikdar S, Banerjee M, Vemparala S (2022a) Role of disulphide bonds in membrane partitioning of a viral peptide. J Membr Biol 255(2):129–142. 10.1007/s00232-022-00218-0 35218393 10.1007/s00232-022-00218-0 PMC8881898

[CR65] Sikdar S, Rani G, Vemparala S (2022b) Role of lipid packing defects in determining membrane interactions of antimicrobial polymers. Langmuir 39(12):4406–4412 10.1021/acs.langmuir.3c00031 36920370

[CR66] Sikdar S, Rani G, Vemparala S (2023) Role of lipid packing defects in determining membrane interactions of antimicrobial polymers. Langmuir 39(12):4406–4412 36920370 10.1021/acs.langmuir.3c00031

[CR67] Søndergaard CR, Olsson MHM, Rostkowski M, Jensen JH (2011) Improved treatment of ligands and coupling effects in empirical calculation and rationalization of pKa values. J Chem Theory Comput 7(7):2284–2295. 10.1021/ct200133y 26606496 10.1021/ct200133y

[CR68] Takahashi H, Caputo GA, Vemparala S, Kuroda K (2017) Synthetic random copolymers as a molecular platform to mimic host-defense antimicrobial peptides. Bioconjug Chem 28(5):1340–1350. 10.1021/acs.bioconjchem.7b00114 28379682 10.1021/acs.bioconjchem.7b00114

[CR69] Tam JP, Wang S, Wong KH, Tan WL (2015) Antimicrobial peptides from plants. Pharmaceuticals 8(4):711–757 26580629 10.3390/ph8040711 PMC4695807

[CR70] Tossi A, Sandri L, Giangaspero A (2000) Amphipathic, α-helical antimicrobial peptides. Pept Sci 55(1):4–30. 10.1002/1097-0282(2000)55:1<4::AID-BIP30>3.0.CO;2-M 10.1002/1097-0282(2000)55:1<4::AID-BIP30>3.0.CO;2-M 10931439

[CR71] Tripathy M, Thangamani S, Srivastava A (2020) Three-dimensional packing defects in lipid membrane as a function of membrane order. J Chem Theory Comput 16(12):7800–7816 33226805 10.1021/acs.jctc.0c00609

[CR72] Uppu DSSM, Samaddar S, Hoque J, Konai MM, Krishnamoorthy P, Shome BR, Haldar J (2016) Side chain degradable cationic-amphiphilic polymers with tunable hydrophobicity show in vivo activity. Biomacromolecules 17(9):3094–3102. 10.1021/acs.biomac.6b01057 27442617 10.1021/acs.biomac.6b01057

[CR73] Vamparys L, Gautier R, Vanni S, Bennett WFD, Tieleman DP, Antonny B, Etchebest C, Fuchs PFJ (2013) Conical lipids in flat bilayers induce packing defects similar to that induced by positive curvature. Biophys J 104(3):585–593. 10.1016/j.bpj.2012.11.3836 23442909 10.1016/j.bpj.2012.11.3836 PMC3566444

[CR20] Van Hilten N, Stroh KS, Risselada HJ (2020) Membrane thinning induces sorting of lipids and the amphipathic lipid packing sensor (ALPS) protein motif. Front Physiol 11:250 32372966 10.3389/fphys.2020.00250 PMC7177014

[CR34] Van Lehn RC, Ricci M, Silva PH, Andreozzi P, Reguera J, Voitchovsky K, Stellacci F, Alexander-Katz A (2014) Lipid tail protrusions mediate the insertion of nanoparticles into model cell membranes. Nat Commun 5(1):1–11 10.1038/ncomms5482 25042518

[CR75] Vanni S, Vamparys L, Gautier R, Drin G, Etchebest C, Fuchs PF, Antonny B (2013) Amphipathic lipid packing sensor motifs: probing bilayer defects with hydrophobic residues. Biophys J 104(3):575–584. 10.1016/j.bpj.2012.11.3837 23442908 10.1016/j.bpj.2012.11.3837 PMC3566459

[CR74] Vanni S, Hirose H, Barelli H, Antonny B, Gautier R (2014) A sub-nanometre view of how membrane curvature and composition modulate lipid packing and protein recruitment. Nat Commun 5:4916 25222832 10.1038/ncomms5916

[CR76] Wang S, Zeng X, Yang Q, Qiao S (2016) Antimicrobial peptides as potential alternatives to antibiotics in food animal industry. Int J Mol Sci. 10.3390/ijms17050603 27153059 10.3390/ijms17050603 PMC4881439

[CR77] Wildermuth KD, Monje-Galvan V, Warburton LM, Klauda JB (2019) Effect of membrane lipid packing on stable binding of the ALPS peptide. J Chem Theory Comput 15(2):1418–1429. 10.1021/acs.jctc.8b00945 30633866 10.1021/acs.jctc.8b00945

[CR78] Wu M, Maier E, Benz R, Hancock RE (1999) Mechanism of interaction of different classes of cationic antimicrobial peptides with planar bilayers and with the cytoplasmic membrane of *Escherichia coli*. Biochemistry 38(22):7235–7242. 10.1021/bi9826299 10353835 10.1021/bi9826299

[CR79] Yang Y, Cai Z, Huang Z, Tang X, Zhang X (2018) Antimicrobial cationic polymers: from structural design to functional control. Polym J 50(1):33–44. 10.1038/pj.2017.72

[CR80] Yeaman MR, Yount NY (2003) Mechanisms of antimicrobial peptide action and resistance. Pharmacol Rev 55(1):27–55. 10.1124/pr.55.1.2 12615953 10.1124/pr.55.1.2

[CR81] Zasloff M (2002a) Antimicrobial peptides of multicellular organisms. Nature 415(6870):389–395. 10.1038/415389a 11807545 10.1038/415389a

[CR82] Zasloff M (2002b) Antimicrobial peptides of multicellular organisms. Nature 415(6870):389–395. 10.1038/415389a 11807545 10.1038/415389a

[CR83] Zhang S-K, Song J-W, Gong F, Li S-B, Chang H-Y, Xie H-M, Gao H-W, Tan Y-X, Ji S-P (2016) Design of an -helical antimicrobial peptide with improved cell-selective and potent anti-biofilm activity. Sci Rep 6(1):27394. 10.1038/srep27394 27271216 10.1038/srep27394 PMC4897634

