# Supplementary material for: Correlation Between Antimicrobial Structural Classes and Membrane Partitioning: Role of Emerging Lipid Packing Defects
Source: J Membr Biol. 2024 Jul 22;257(5-6):307–21. doi: 10.1007/s00232-024-00318-z (PMC11584508; doi:10.1007/s00232-024-00318-z)
Supplement: Supplementary file 1 — (pdf 22059 KB) [file 232_2024_318_MOESM1_ESM.pdf]

Supporting Information

Correlation between antimicrobial structural classes and membrane partitioning: Role of emerging lipid packing defects

S V Sankaran,[1, 2, *] Roni Saiba,[1, 2, †] Samapan Sikdar,[1, 2, ‡] and Satyavani Vemparala[1, 2, §]

[1] *The Institute of Mathematical Sciences, C.I.T. Campus, Taramani, Chennai 600113, India*
[2] *Homi Bhabha National Institute, Training School Complex, Anushakti Nagar, Mumbai, 400094, India*

(Dated: May 26, 2024)

* mailsankaran.sv@gmail.com
† ronis@imsc.res.in
‡ samapan@imsc.res.in
§ vani@imsc.res.in

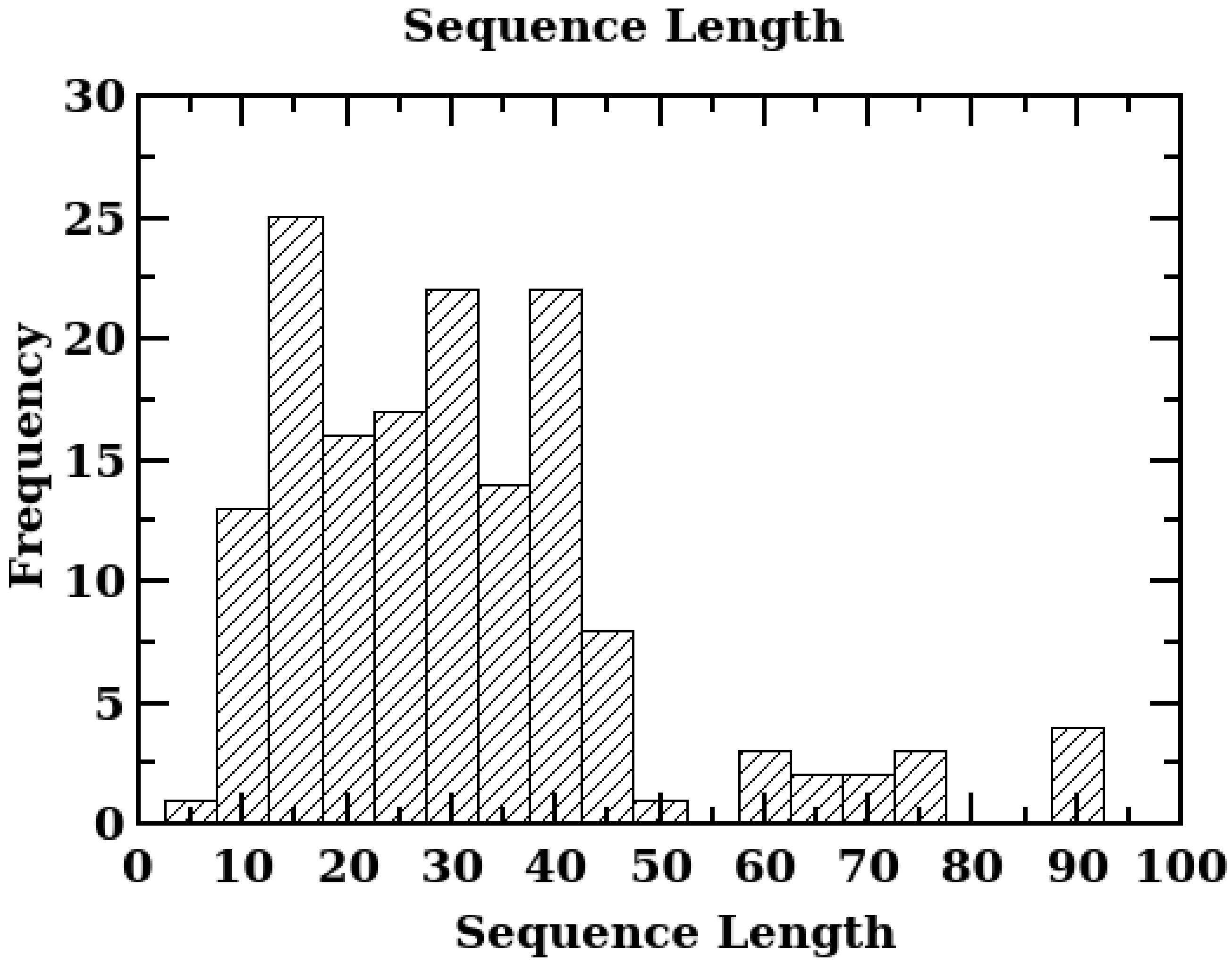

FIG. S.1. Distribution of sequence lengths of AMPs in DRAMP data base.

TABLE S.1. MD simulation details

Peptide	Simulation time -Replica 1 (ns)	Simulation time -Replica 2 (ns)
2B5B	345	293
2EEM	400	429
2JSB	368	474
2MMM	400	417

TABLE S.2. MD system details- Replica 1

Peptide	Simulation time -Replica 1 (ns)	Initial box size (Å^3)	Total atoms	Lipid atoms	Water atomss
2B5B	345	110 x 110 x 110	91379	22500	58470
2EEM	400	110 x 110 x 80	67044	22500	43873
2JSB	368	110 x 110 x 85	63313	22500	30639
2MMM	400	110 x 110 x 105	78979	22500	46104

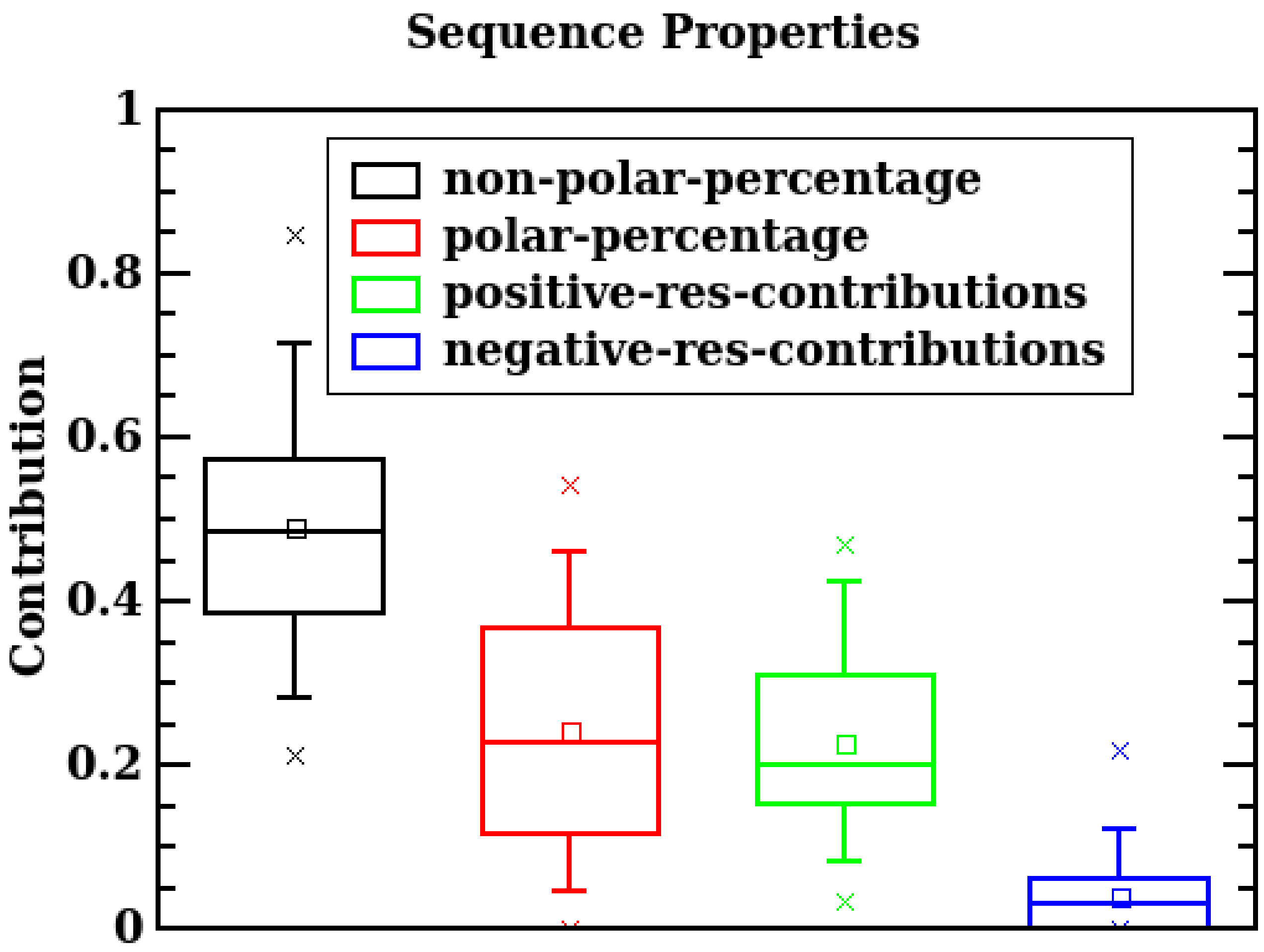

FIG. S.2. Box plot of distribution of different classes of residues of AMPs in DRAMP data base

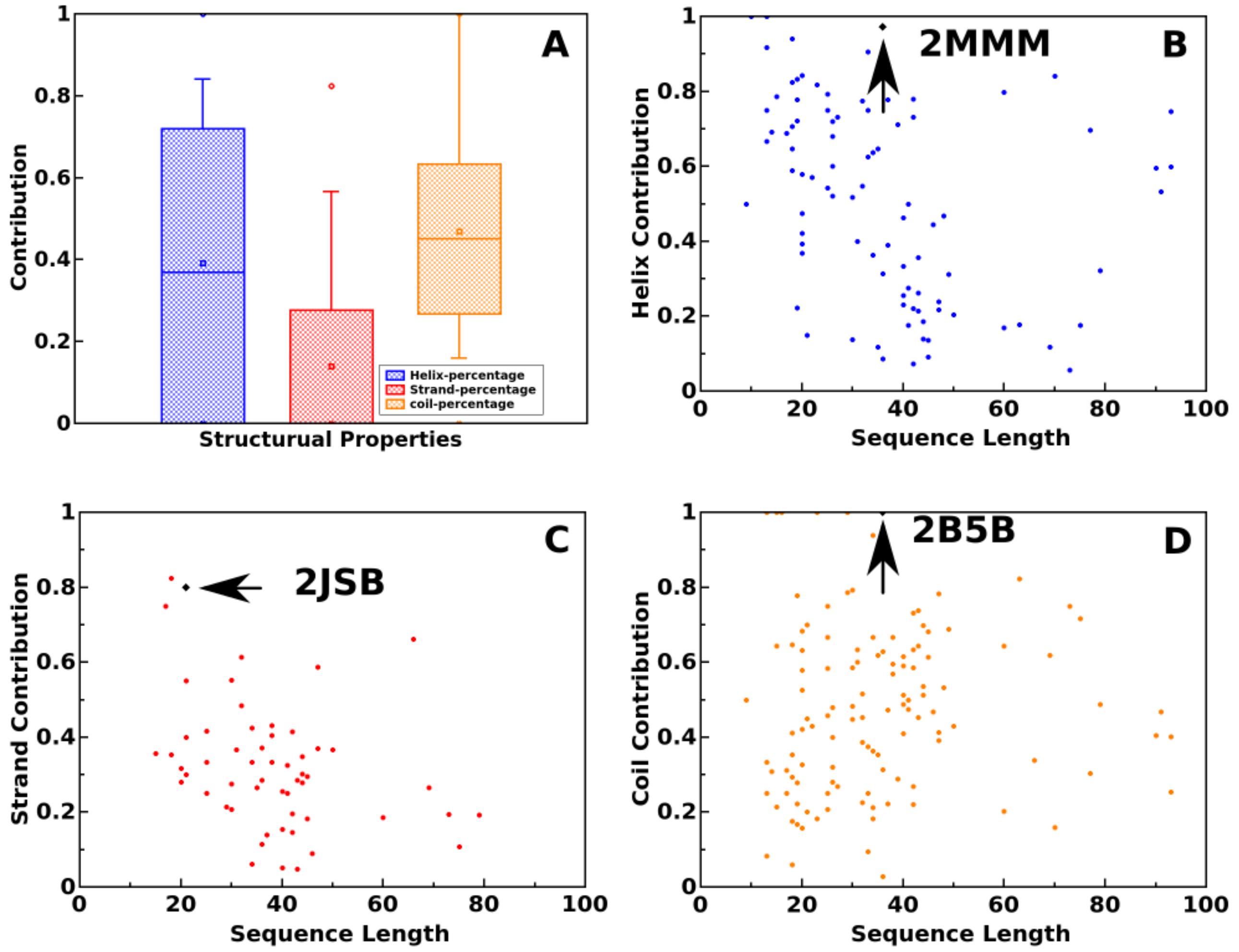

FIG. S.3. A) The box plot showing the contribution from all the 3 structural properties. The dots in the points in the center, top and bottom correspond to the mean, maximum and minimum values of the secondary structural percentage values. B) Sequence length vs helix contribution. C) Sequence length vs strand contribution. D) Sequence length vs coil contribution

PDB_ID	sequence_length	Helix_percentage	Strand_percentage	coil_percentage			
2N0V	9	0.5	0	0.5			
1N6T	10	1	0	0			
1VM2	13	1	0	0			
1VM5	13	0.917	0	0.083			
1VM4	13	0.917	0	0.083			
2MAA	13	0.75	0	0.25			
6GS5	13	0.667	0	0.333			
1HR1	13	0.667	0	0.333			
1QXQ	13	0	0	1			
1D6X	13	0	0	1			
6GIL	13	0	0	1			
2DD6	13	0	0	1		**Choosen structures**	
1G8C	13	0	0	1			
1D7N	14	0.692	0	0.308	**2MMM**		Helix
2JMY	15	0.786	0	0.214	**2JSB**		Strand
2N5C	15	0	0.357	0.643	**2EEM**		Helix + strand
1QX9	15	0	0	1	**2B5B**		Coil
2MXG	16	0	0	1			
1F0F	17	0.688	0	0.312			
1WO0	17	0	0.75	0.25			
2N0O	18	0.941	0	0.059			
1HU5	18	0.824	0	0.176			
2RLH	18	0.824	0	0.176			
1HU6	18	0.706	0	0.294			
1F0E	18	0.647	0	0.353			
1HU7	18	0.588	0	0.412			
1HVZ	18	0	0.824	0.176			
1RKK	18	0	0.353	0.647			
2JQ0	19	0.833	0	0.167			
2JPY	19	0.833	0	0.167			
1P0J	19	0.833	0	0.167			
1P0L	19	0.833	0	0.167			
1P0O	19	0.833	0	0.167			
1P5K	19	0.778	0	0.222			
1P5L	19	0.778	0	0.222			
2JQ1	19	0.722	0	0.278			
1P0G	19	0.722	0	0.278			
5YKL	19	0.222	0	0.778			
5YKQ	19	0.222	0	0.778			
2AP7	20	0.842	0	0.158			
1F0D	20	0.579	0	0.421			
5X3L	20	0.474	0	0.526			
1F0H	20	0.421	0	0.579			
4EZN	20	0.393	0.28	0.327			
1F0G	20	0.368	0	0.632			
1M4E	20	0	0.316	0.684			
8TFV	21	0.15	0.4	0.45			
2JSB	21	0	0.8	0.2			
2JNI	21	0	0.8	0.2			
1Q71	21	0	0.55	0.45			
1S6W	21	0	0.3	0.7			

2JOS	22	0.571	0	0.429
2L5R	23	0.818	0	0.182
2MAG	23	0.818	0	0.182
2LTI	23	0	0	1
1Z64	25	0.792	0	0.208
2KHF	25	0.75	0	0.25
5H2S	25	0.75	0	0.25
1ZRW	25	0.542	0	0.458
1CZ6	25	0	0.417	0.583
1M4F	25	0	0.333	0.667
1LFC	25	0	0.25	0.75
2G9P	26	0.72	0	0.28
2PCO	26	0.68	0	0.32
2AMN	26	0.68	0	0.32
1BH1	26	0.6	0	0.4
2KET	26	0.6	0	0.4
1YTR	26	0.52	0	0.48
2MHW	27	0.731	0	0.269
2KHB	29	0	0.214	0.786
1FRY	29	0	0	1
2LR7	29	0	0	1
2L3I	30	0.517	0	0.483
2MLU	30	0.517	0	0.483
1MMC	30	0.138	0.276	0.586
1DFN	30	0	0.552	0.448
1BH4	30	0	0.207	0.793
2KHT	30	0	0.207	0.793
2N92	31	0.4	0	0.6
2ERI	31	0	0.367	0.633
2K10	32	0.774	0	0.226
2KEG	32	0.548	0	0.452
1ZMP	32	0	0.613	0.387
1ZMQ	32	0	0.613	0.387
2K1I	32	0	0.484	0.516
1EWS	32	0	0.484	0.516
2K9B	33	0.906	0	0.094
1XC0	33	0.75	0	0.25
2JUI	33	0.625	0	0.375
2RLW	34	0.818	0	0.182
2MWT	34	0.636	0	0.364
2EEM	34	0.364	0.424	0.212
1TYK	34	0	0.333	0.667
2MXQ	34	0	0.061	0.939
2K38	35	0.647	0	0.353
2JPK	35	0.647	0	0.353
1E4R	35	0.118	0.265	0.618
2MMM	36	0.971	0	0.029
1MM0	36	0.314	0.371	0.314
2LG5	36	0.086	0.286	0.629
1IYC	36	0	0.114	0.886
2B5B	36	0	0	1
2G9L	37	0.778	0	0.222

1CW6	37	0.389	0.139	0.472				
1UT3	38	0	0.432	0.568				
1BNB	38	0	0.405	0.595				
1DKC	38	0	0.333	0.667				
2JPJ	39	0.711	0	0.289				
2LG4	40	0.462	0.051	0.487				
1L4V	40	0.333	0.256	0.41				
2NZ3	40	0.256	0.154	0.59				
1ZFU	40	0.231	0.256	0.513				
2NY8	40	0.231	0.154	0.615				
2A2B	41	0.5	0	0.5				
2NY9	41	0.275	0.25	0.475				
1FQQ	41	0.175	0.325	0.5				
1ZRX	42	0.78	0	0.22				
2JR8	42	0.732	0	0.268				
1KV4	42	0.732	0	0.268				
1X22	42	0.732	0	0.268				
1Z99	42	0.22	0.146	0.634				
2JR3	42	0.073	0.195	0.732				
2DCV	42	0	0.415	0.585				
2DCW	42	0	0.415	0.585				
1OHN	43	0.357	0	0.643				
2B68	43	0.262	0.286	0.452				
2E3F	43	0.214	0.048	0.738				
1I2V	44	0.186	0.279	0.535				
2LB7	44	0.14	0.349	0.512				
1CIX	44	0	0.302	0.698				
2E3E	45	0.136	0.182	0.682				
1KJ6	45	0.091	0.295	0.614				
3C8P	46	0.444	0.089	0.467				
2GL1	47	0.239	0.37	0.391				
1XV3	47	0.217	0	0.783				
2B9K	47	0	0.587	0.413				
1CW5	48	0.468	0	0.532				
1Z6V	49	0.312	0	0.688				
1BK8	50	0.204	0.367	0.429				
2KJF	60	0.797	0	0.203				
2K35	60	0.169	0.186	0.644				
1UEO	63	0.177	0	0.823				
2LGN	66	0	0.662	0.338				
1QNK	69	0.118	0.265	0.618				
1DQC	73	0.056	0.194	0.75				
2LN8	75	0.176	0.108	0.716				
1OF9	77	0.697	0	0.303				
2RNG	79	0.321	0.192	0.487				
2KNJ	90	0.596	0	0.404				
1SIY	91	0.533	0	0.467				
1MR8	93	0.746	0	0.254				
2MAL	93	0.598	0	0.402				

FIG. S.4. PDB Ids and secondary structural content. The representative PDBs selected for molecular dynamics simulations are highlighted, based on optimal secondary structural propensity

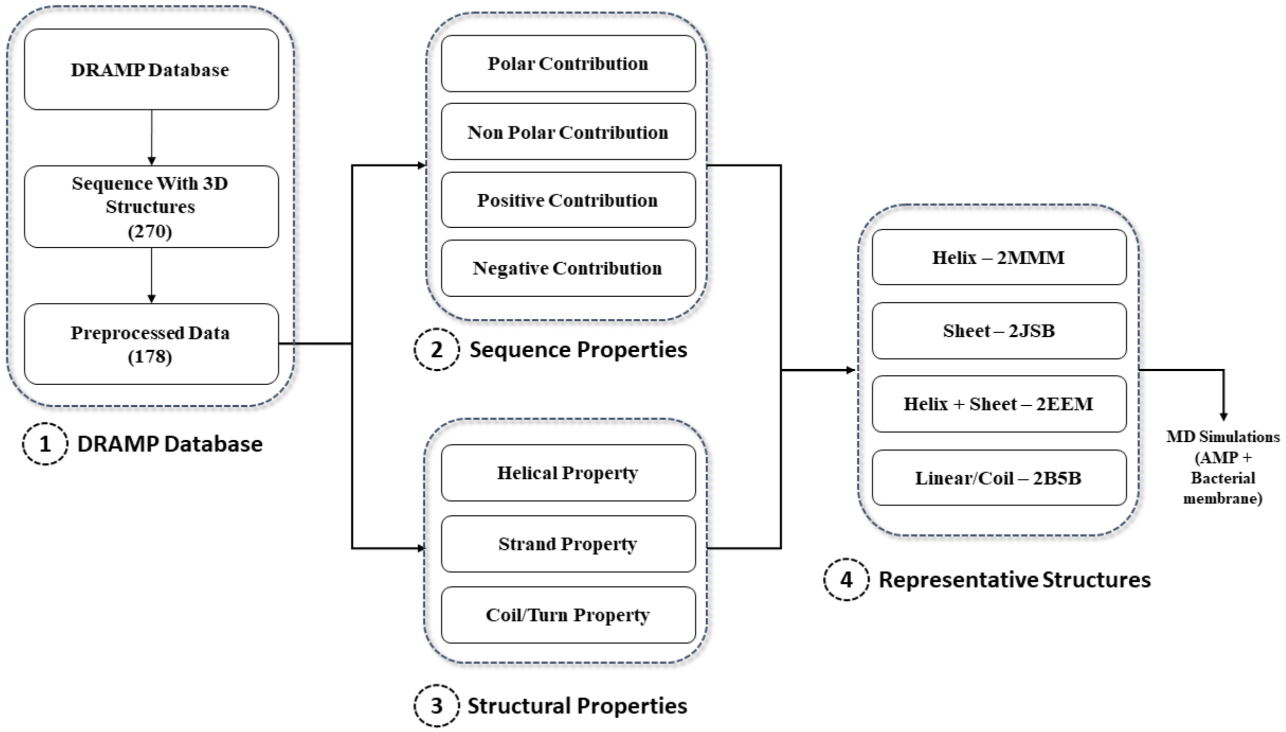

FIG. S.5. Analyses and simulation pipeline used in this study

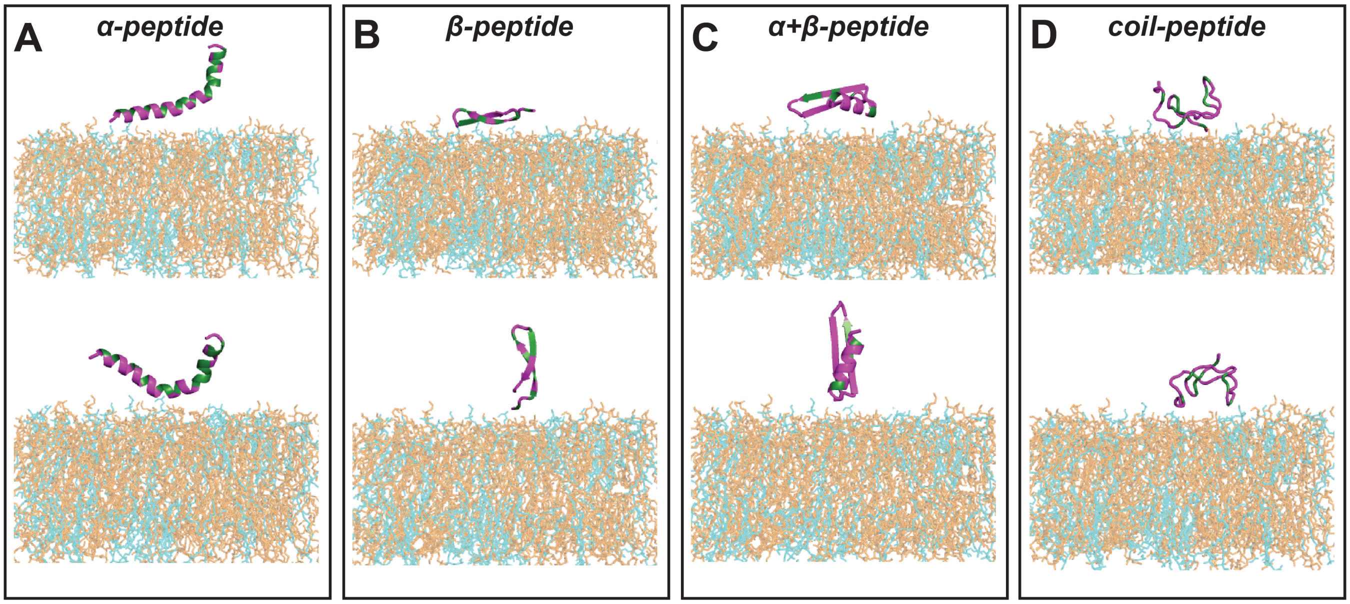

FIG. S.6. Initial setup of four AMPs on a model bacterial along the membrane normal are shown. Water and ions are not shown for clarity. The hydrophobic and hydrophilic residues of AMPs are coloured in green and pink respectively. The POPE and POPG lipid molecules in the membrane are coloured as orange and cyan respectively.

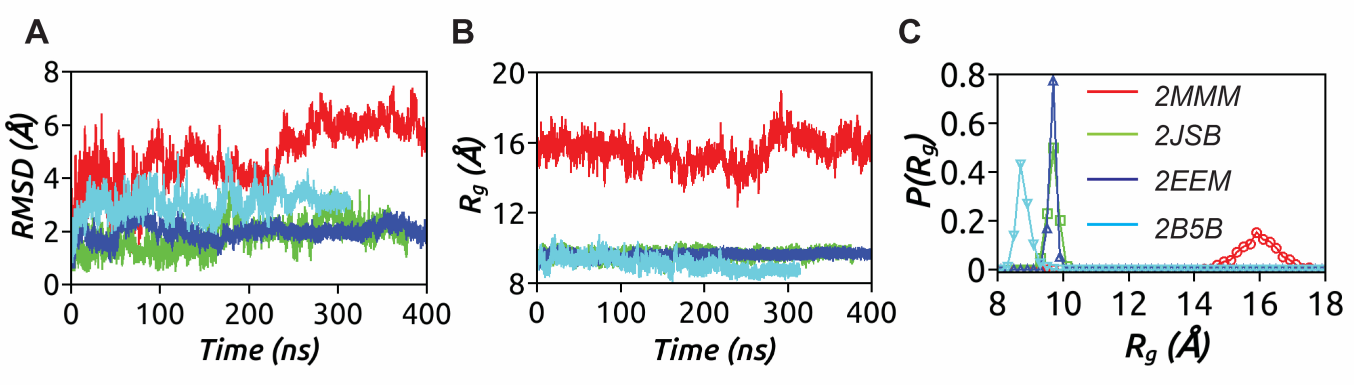

FIG. S.7. Structural properties of simulated four AMPs in the bilayer environment. (a) root mean squared deviation from the initial solution crystal structures (b) radius of gyration indicating the size of the AMPs (c) conformational sampling of the AMPs, in terms of R_g, to understand changes in the structures as the membrane simulation progresses.

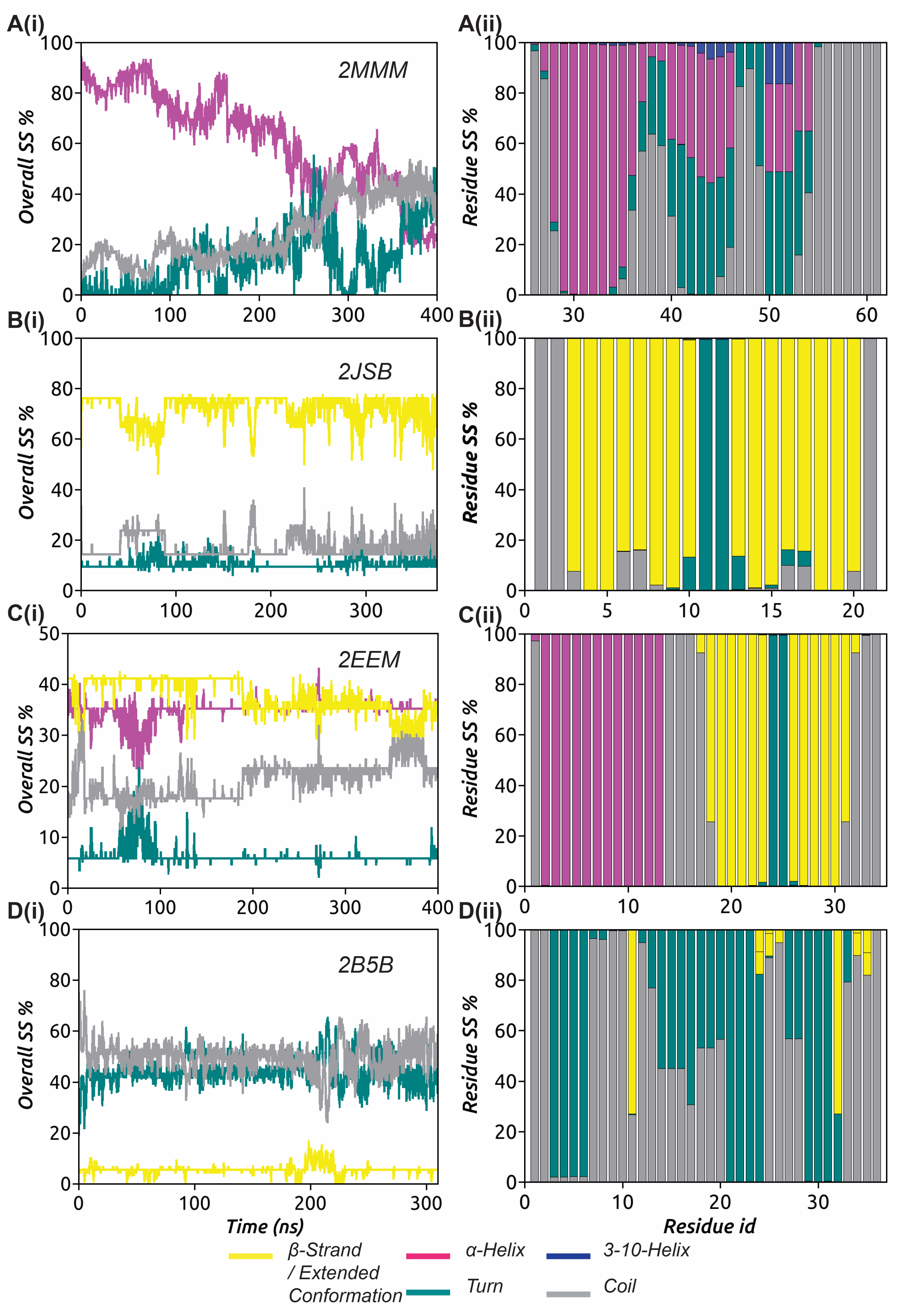

FIG. S.8. Evolution of secondary structural elements in the four AMPs during the interaction with the model bacterial membrane are shown.

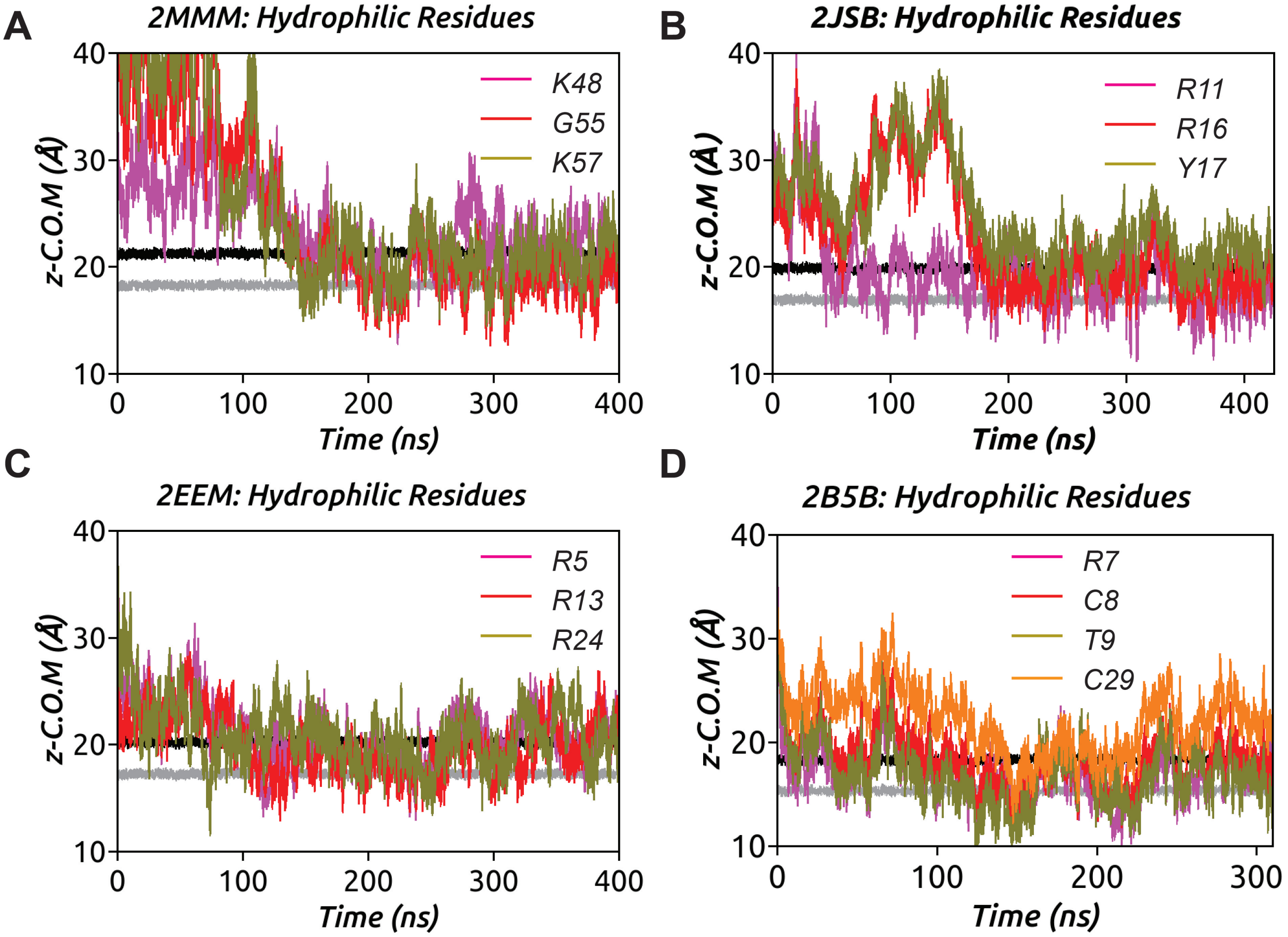

FIG. S.9. Partitioning of key hydrophilic residues into the model bacterial membrane during the simulation for four AMPs considered. The locations of lipid headgroup P atoms (black) and C2 atoms (grey) of glycerol moieties of the upper leaflet, along the membrane normal, of the model bacterial membrane are also shown.

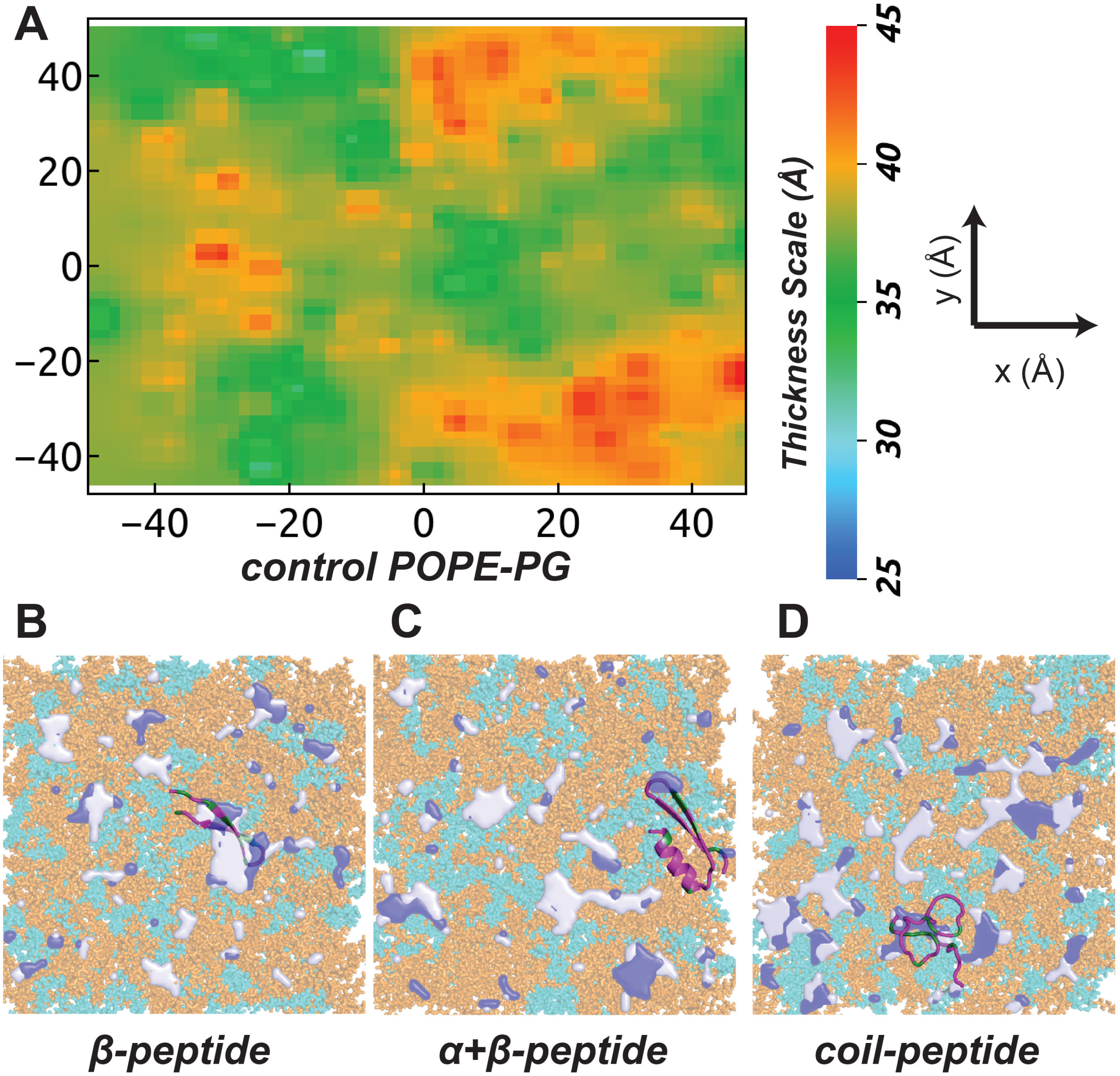

FIG. S.10. (A) The 2D-thickness map based on inter-leaflet P-P distance generated using 2X2 Å resolution along the x-y plane for the final MD snapshot of control POPE-PG model bacterial membrane without any AMPs. The scale remains the same as for the AMP-membrane systems considered in this study. (B-D) The final snapshots of AMPs-POPE (orange)/PG (cyan) system illustrate deep (dark blue) and shallow defect (light blue) sites.
